# Assessment of forest cover and carbon stock changes in sub-tropical pine forest of Azad Jammu & Kashmir (AJK), Pakistan using multi-temporal Landsat satellite data and field inventory

**DOI:** 10.1371/journal.pone.0226341

**Published:** 2020-01-23

**Authors:** Iftikhar Ahmad Khan, Mobushir Riaz Khan, Muhammad Hasan Ali Baig, Zaker Hussain, Nasir Hameed, Junaid Aziz Khan

**Affiliations:** 1 Department of Forest, Azad Jammu & Kashmir, Pakistan; 2 Institute of Geo-information & Earth Observation (IGEO), PMAS-Arid Agriculture University Rawalpindi, Pakistan; 3 Land Use Planning Section, Planning & Development Department, Azad Jammu & Kashmir, Pakistan; 4 Institute of Geographic Information System, National University of Science & Technology- Islamabad, Pakistan; Assam University, INDIA

## Abstract

This study aimed at estimating temporal (1989–2018) change in forest cover, carbon stock and trend in corresponding CO_2_ emissions/sequestration of a sub-tropical pine forest (STPF) in AJK, Pakistan. Our field inventory estimation shows an average above ground biomass (AAGB) accumulation of 0.145 Kt/ha with average carbon stock (ACS) value of 0.072 Kt/ha. Landsat TM, ETM+ and OLI images of 1989, 1993, 1999, 2005, 2010, 2015 and 2018 were used to extract vegetation fractions through Linear Spectral Mixture Analysis (LSMA) and forest area was calculated for respective years. Based on the forest area and estimated ACS value, the biomass carbon stock with corresponding CO_2_ emissions/sequestration was worked out for each time and change in forest carbon stock was determined for different time periods from 1989 to 2018. Our analysis shows net increase of 561 ha in forest cover and 40.39 Kt of ACS along with increase in corresponding CO_2_ sequestrations of 147.83 Kt over the study period. The results based on combination of remote sensing and field inventory provide valuable information and scientific basis to plan and ensure sustainable forest management (SFM) through reforestation, protection and conservation to enhance and maintain adequate forest cover and reduce CO_2_ emissions.

## 1. Introduction

The global terrestrial ecosystem encompass forests as major component which cover around 31% of earth’s land surface and the area under forest cover is considered as an important indicator of environmental condition [1]. Forests play a vital role in photosynthetic alleviation of atmospheric CO_2_ and its long-term storage as wood biomass [2] which contains approximately 50% of carbon [3]. Conversely, the stored carbon is released into the atmosphere as carbon dioxide (CO_2_) when forests are cleared or degraded [4]. Hence, deforestation is responsible for approximately 20% of anthropogenic emissions worldwide [5, 6] with varying regional statistics [7]. Globally, a net decrease of 1.7% in the forest area with an annual rate of change of 0.11% was reported for the period 1990–2005 [8] and it was estimated that during the period 2005–2010 the reduction in global forest area led to decrease in forest biomass carbon stock by an amount of 0.5 Gt annually [7]. Sustainable forest management (SFM) greatly relies on the important components like assessments of forest cover, forest carbon stocks and carbon emissions from deforestation and degradation [9]. Forest resource estimation and its intervallic change assessment are important dynamics which drive forest inventories aimed at SFM. The scientific quantification of forest cover and temporal changes provide valuable information to expose deforestation or appreciate successful reforestation programmes at particular sites and allows for appropriate land use planning along with carbon accounting and monitoring of conservation efforts [10, 11]. Also, accurate and timely information regarding vegetation changes is a prerequisite to resource managers and policy makers [12] for future planning and predictions. The technological advancement has enabled scientists to develop different methods for detecting changes in land and vegetation cover over the last 30 years [13]. Many forest cover related studies [14, 15, 16, 17, 18, 19] have used satellite images to retrieve fractional information embedded in pixels through a widely used technique called SMA which helps address mixed pixel problem [20].

In recent times, remote sensing has widely been used to detect temporal changes in land cover and make it an effective tool on account of its digital data format and consistent coverage of land cover at varying resolutions. Moreover, it enables to capture continuous, precise and impartial information about spatial variability of land surface features that becomes quickly available for use [21, 22]. In present era, remote sensing is an effective mode of collecting forest cover information [23, 24] for practical and economical study of vegetation cover changes, particularly over large landscapes [25, 26]. Nevertheless, its usefulness in providing meaningful data regarding growing stock, forest area and forest land use change is well established for developing forest inventory [27]. In this context, Landsat provides long history of dataset archive which is vital in mapping long-term vegetation cover and studying spatiotemporal changes [28] because Landsat data with fine/high spatial resolutions are recommended for reliable quantification of forest cover change.

The State of Azad Jammu & Kashmir (AJK) is blessed with high valued natural fauna stretching along Himalayan mountainous topography classified as Subtropical, Temperate and Alpine forests. The predominant coniferous species include *Pinus roxburghii*, *Pinus wallichiana*, *Abies pindrow* and *Cedrus deodara*. These forests are being managed under traditional inventories which lack critical scientific information on carbon stock density and temporal land cover changes. The objective of this case study is the estimation of ACS per hectare in a subtropical pine forest using field inventory and assessment of historical stock change patterns through periodic analysis of satellite imageries (1989–2018). The findings will help draw a comparison between deforestation and the overall success of reforestation programmes implemented in the area over the study period. The study will also enable forest managers to develop forest inventories on the basis of modern scientific parameters to ensure SFM.

## 2. Materials and methods

This section describes the study area, method of field inventory and satellite data acquisition and processing.

### 2.1 Study area

**Fig 1:**
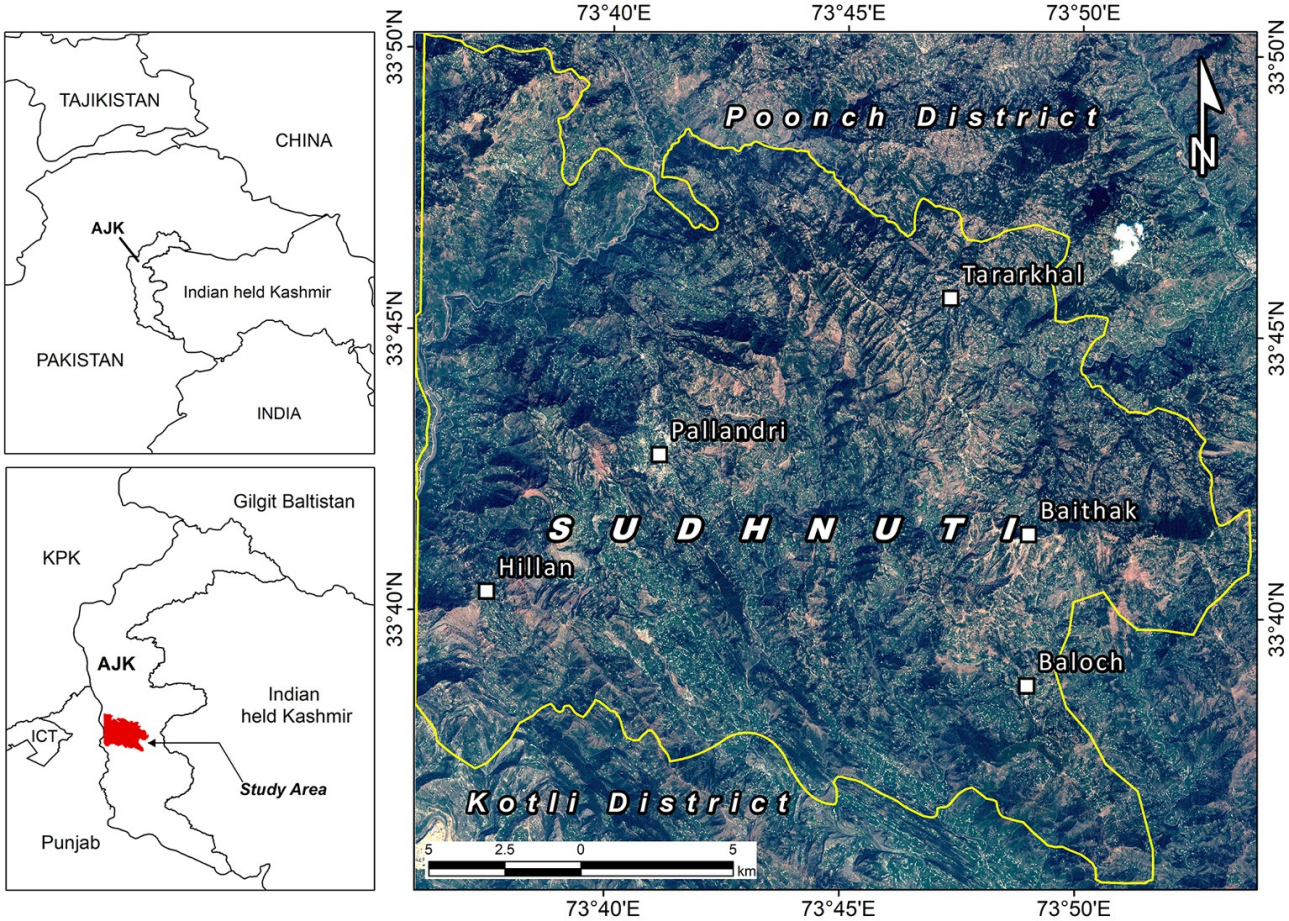
Study area map.

The study area (471 km^2^) lies between 73°35'45.49"E, 33°50'14.24"N to 73°53'44.84"E, 33°35'19.03"N in district Sudhnuti AJK, Pakistan (Fig 1). The elevation ranges from 385 m to 2121 m ASL with maximum slope up to 78 degree and mean annual temperature varies from 110C and 250C having 1432 mm average rainfall. The predominant species is Chir Pine *(Pinus roxburghii)* with a little mix of Blue Pine *(Pinus wallichiana)* when approaching temperate forest [29]. It is managed by the State Forest Department. Distribution of vegetation is fragmented across the landscape.

### 2.2 Field inventory & forest carbon stock assessment

**Fig 2:**
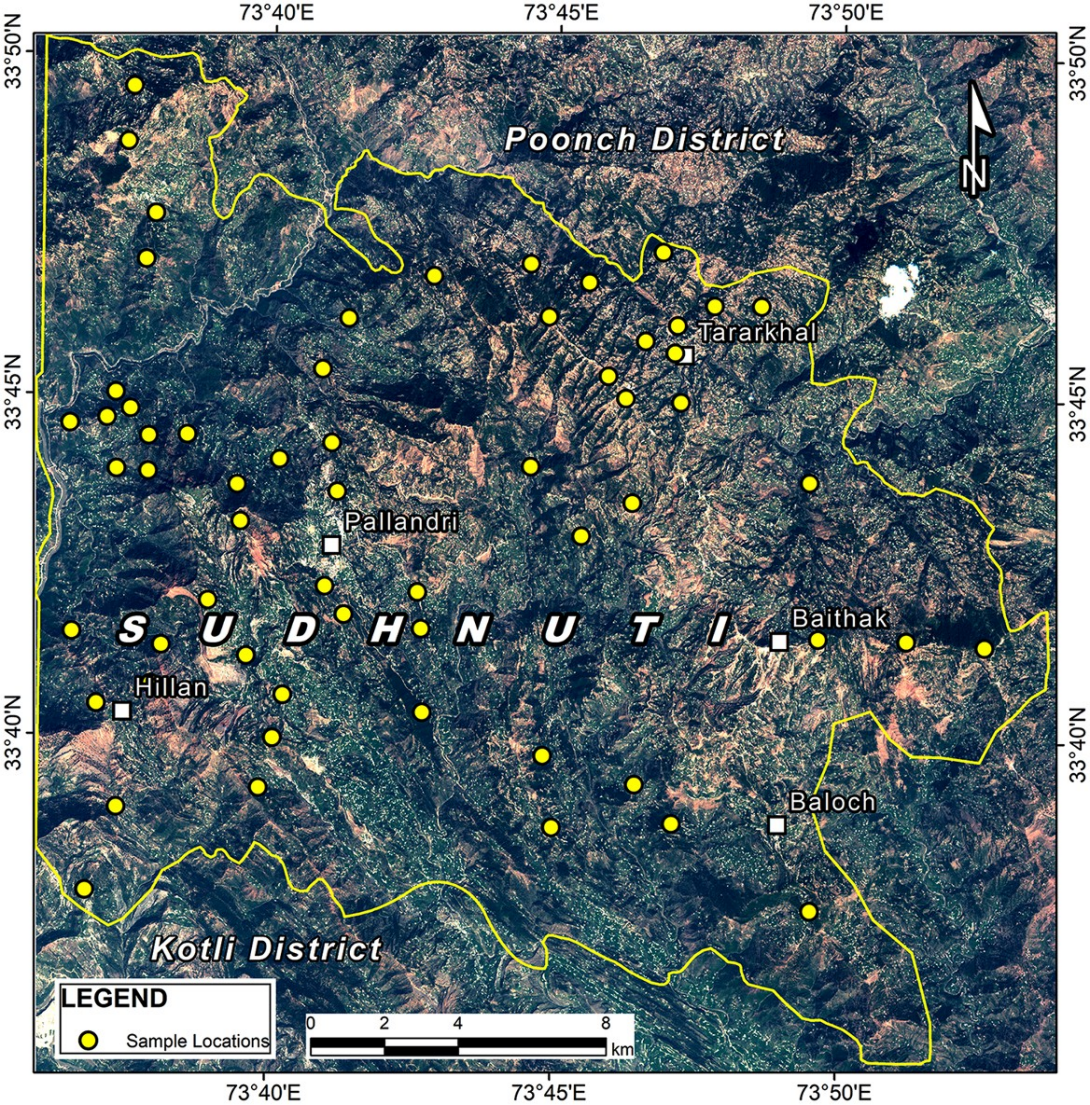
Distribution of sample locations.

A pilot survey of the study area accompanied by field staff of the respective forest division having knowledge of the area and forest distribution was conducted to collect preliminary data to determine 108 circular sample plots across study area (Fig 2)using the source book for “Land Use, Land Use Change and Forestry Projects” [30] and detailed inventory was carried out during January-July, 2017 (S1 Fig and S1 File). Field inventory was carried out with the permission of Chief Conservator of Forests (CCF), Government of the State of AJK (GoAJK). The study area did not include any endangered flora. GPS was used to locate each sample plot of 0.1 ha on ground while slope was determined using clinometer to adjust the plot radius accordingly (S2 Fig). Sampling was conducted for trees having minimum 15cm diameter at breast height (dbh) to match the criteria laid down for management plans of the State forests. The tree height and diameter (dbh) were measured using Abney Level and Caliper respectively (S2 Fig). The area specific allometric equations developed by Pakistan Forest Institute (PFI) were retrieved from technical reports to calculate AGB of *Pinus roxburghii*, *Pinus wallichiana* and *Quercus incana* being the predominant species of the study area. Average below ground biomass (ABGB) was calculated using the relationship between AGB and BGB. A conversion factor of 0.5 [31, 32] was used to work out the forest biomass carbon while total average biomass (t/ha) was worked out by adding AGB and BGB. Average above ground biomass (AAGB) value was used for estimation of temporal stock changes and corresponding CO_2_ emissions/sequestrations.

### 2.3 Satellite data collection and pre-processing

The study involved Level-1 Precision and Terrain (L1TP) corrected Landsat data that are considered suitable for time series analysis (www.landsat.usgs.gov*)*. Landsat 5 Thematic Mapper (TM), Landsat 7 Enhanced Thematic Mapper Plus (ETM+) and Landsat 8 Operational Land Imager (OLI) sensors data corresponding to the study area were downloaded from earthexplorer.usgs.gov (Table 1).

**Table 1:** Landsat satellite images used in the study.

Path/Row	Sensor	Acquisition Date	Scene Cloud Cover (%age)	Scene Quality
149/037	TM	26 December 1989	20	9*
	TM	05 December 1993	1	9
	ETM+	28 November 1999	1	9
	ETM+	30 December 2005	2	9
	TM	20 December 2010	3	9
	OLI	18 December 2015	4.49	9
	OLI	24 November 2018	7.71	9

According to lta.cr.usgs.gov Acquisition Quality 9* = Excellent (no quality issues or errors detected)

Since appropriate selection of imagery acquisition dates is crucial to minimize chances of encountering unwanted changes induced by differences in sun angles or seasons, images with close anniversary dates with minimal/no cloud cover were chosen for this study. Particularly, presence of healthy green grasses along the patches of targeted evergreen vegetation feature widely intermingled across the landscape during summer thus making the two spectrally alike land cover features hard to delineate. The selection of winter season imagery was expected to improve discrimination of targeted vegetation from soil which becomes exposed due to recession of healthy green grasses and deciduous trees during this time of the year.

All the images were co-registered to ensure sub-pixel geometric alignment to minimize errors produced by miss registration of images during time series analysis. The radiometric calibration was performed to compute radiance images which served as input to Fast Line-of-sight Atmospheric Analysis of Hypercubes (FLAASH) module used for atmospheric correction of downloaded data in ENVI 5.3. Later on, spatial sub setting was applied to resultant geometrically and atmospherically preprocessed multi-temporal surface reflectance data to limit the subsequent analysis within study area extent.

### 2.4 Image processing & Spectral Mixture Analysis

Spectral Mixture Analysis (SMA) is one of the most widely used remote sensing methods employed to derive fraction covers from mixed pixels [33]. It helps in accurate extraction of quantitative subpixel information [34]. Spectral “unmixing” can be a linear or nonlinear approach. Linear mixture model assumes that every pixel in the image is a mixture of different spectra (known as endmembers) and sensor recorded spectrum is a linear combination of these spectra [35]. The SMA model assumes that image spectra are formed by linear combinations of n pure spectra [36] and expressed as;

$$R_{b}=\sum_{i=1}^{n}F_{i}\;R_{i,b}+\varepsilon_{b}$$

$$\sum_{i=1}^{n}F_{i}=1$$

*R*_*b*_ is the reflectance in band b, *R*_*i*,*b*_ is the reflectance for endmember i, in band b, *F*_*i*_ is the fraction of endmember i, and *ε*_*b*_ is the residual error for each band. The SMA model error is estimated for each image pixel by computing the Root Mean Square (RMS).

The endmembers used for spectral unmixing may be reference or library endmembers measured in a laboratory or in field conditions or derived from image itself as image endmembers [37]. As the radiometric and atmospheric correction of the images reduces potential noise, therefore, extraction of endmembers from the calibrated data is preferred [38]. On the contrary, endmembers derived from the laboratory spectra requires a great deal of effort [19].

In this study, soil and vegetation were defined as two endmembers [39]. Then samples of these endmembers were visually selected from the scene by identifying the representative areas of each component based on the knowledge of study area [16]. Vegetation and soil fraction images were derived through independent Linear Spectral Unmixing of Landsat scenes involved in the study. The output composite RGB images, each containing fractional cover of soil, vegetation and associated RMS error image, were visually interpreted to ascertain optimal threshold for separation of pure pixels representing targeted vegetation from soil and other features. The RMS error image provides information about areas of missing or incorrect endmembers.

The pixel value of fraction raster derived by SMA provided essential information about fraction of pixel that contains the endmember material related to the image. When individually added, pixels with brighter tone denote higher fraction values, while darker pixels correspond smaller fraction values of endmember material within each pixel. For example, a pixel value of 0.65 indicates that 65% of the pixel contains endmember material. We used thresholding approach for classification of extracted vegetation fractions (VFs) and classification was refined through iterative process [35]. The threshold values of >0.82, >0.73, >0.72, >0.80, >0.81, >0.83 and >0.82were used for classification of VF images of 1989, 1993, 1999, 2005, 2010, 2015 and 2018 respectively. A false color RGB fraction image corresponding to each fraction image was produced with Red color assigned to inherent vegetation fraction and Green and Blue colors were designated to soil fraction band. Resultantly, targeted vegetation pixels with unique red color became apparent and easily distinguishable from rest of the features, specifically surrounding soil (which included bare soil, and built-up).

**Fig 3:**
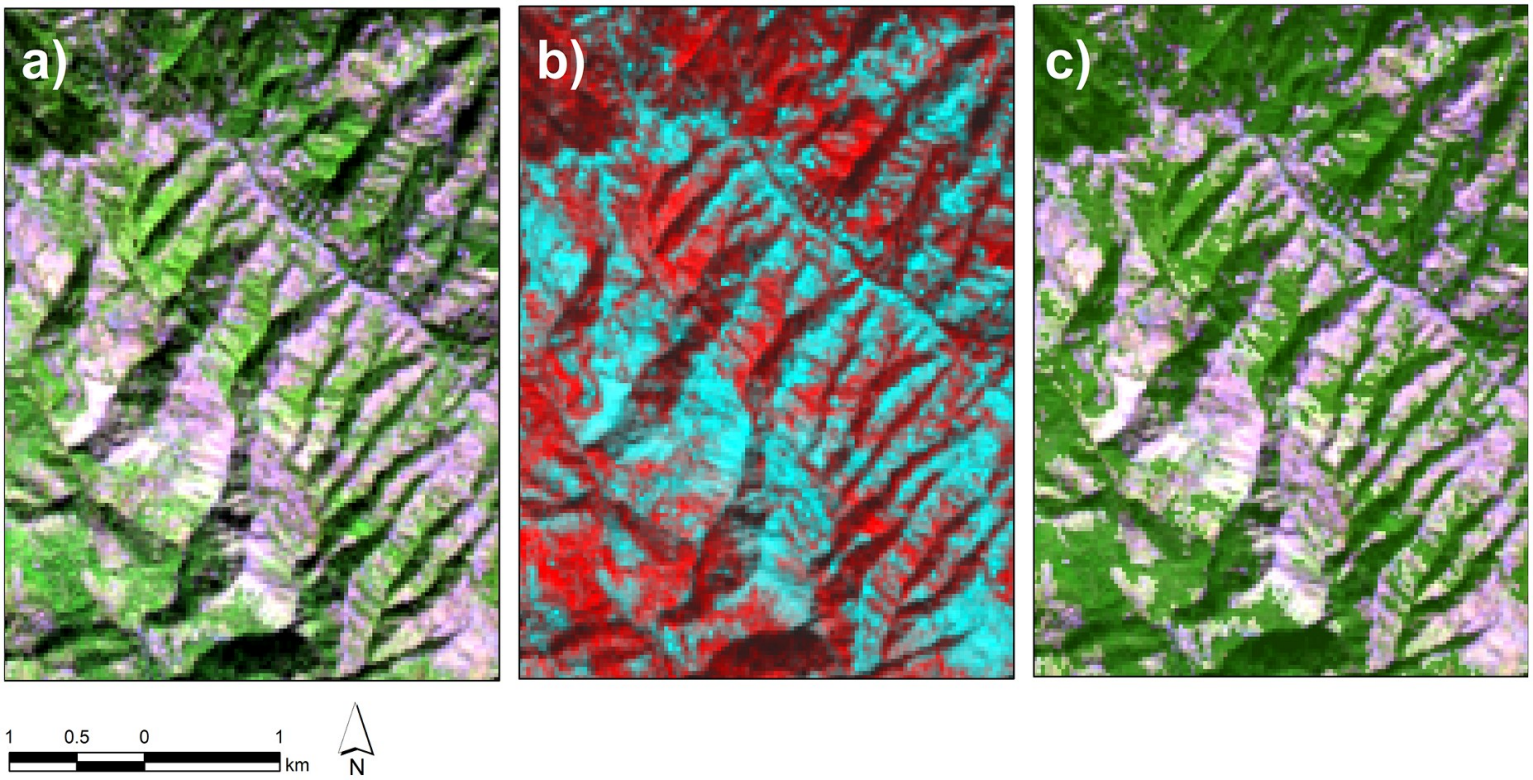
(a). A subset of false color (6,5,4) Landsat 8 OLI image (2018) highlights vegetative areas in green. (b). A composite false color vegetation fraction raster (2108) with customized band combination highlights vegetation in red. (c) Vegetation fraction derived after assigning threshold value through visual interpretation of LSMA raster overlaid on false color composite of corresponding Landsat image.

The false color fraction image considerably facilitated better identification of pixels with abundance of vegetation and assisted in effective selection of representative threshold for its separation from distinct soil feature. An associated false color Landsat image was also produced to further assist the interpretability of the fractional cover. For Landsat TM and ETM+, a band combination of 5,4,3 for Red, Green and Blue color guns respectively, helped highlighting vegetation feature present within the image. While, corresponding band combination for Landsat OLI (6,5,4) was chosen to create false color image to serve the same purpose (Fig 3).

**Fig 4:**
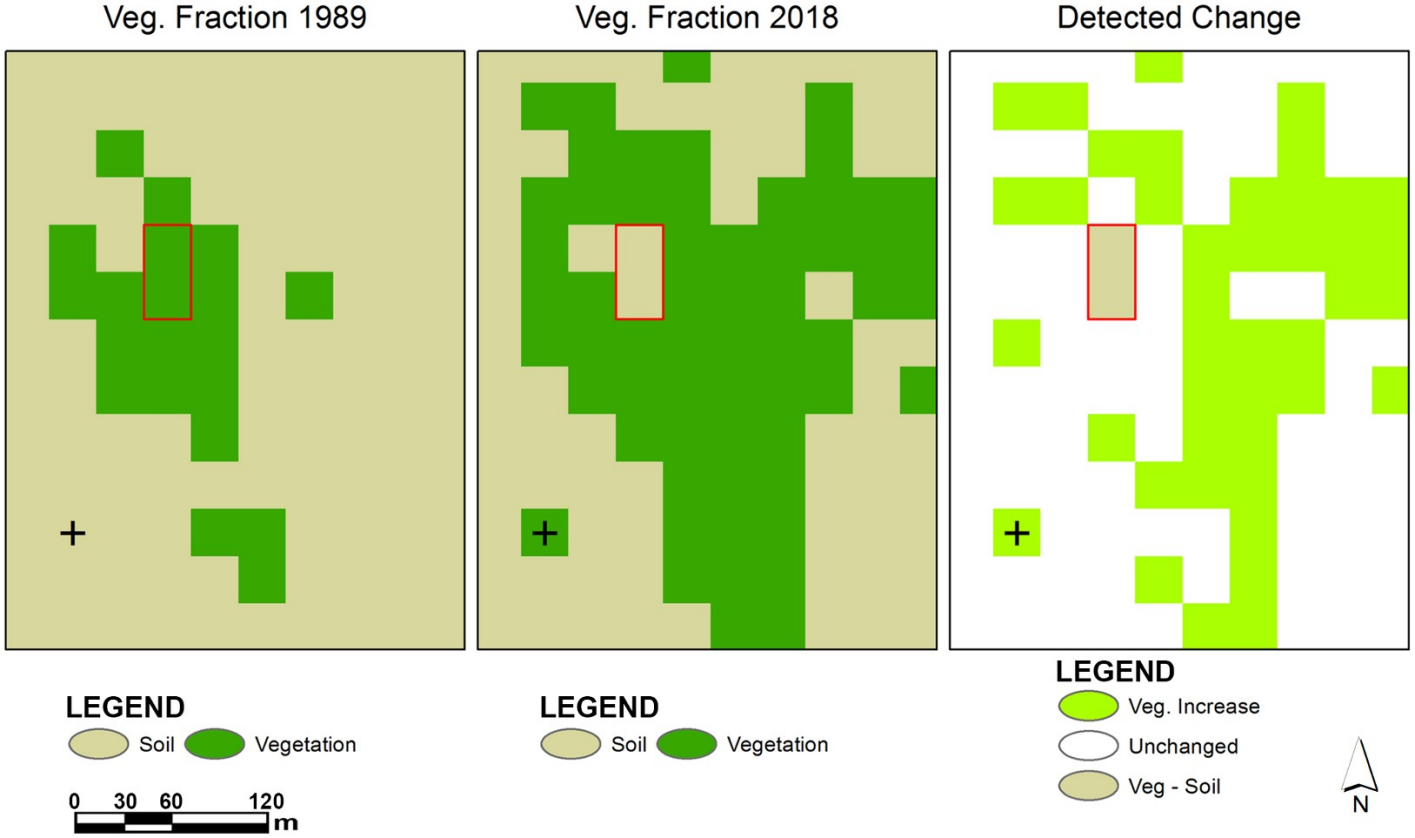
A subset of raster derived from image overlay analysis indicates changes in vegetation cover (1989–2018).

This approach facilitated the thresholding process a great deal and it was further refined iteratively. Following this method, all the derived vegetation fractions were categorized into forest and non-forest cover classes. The reclassified VFs were converted into binary raster format A pixel value of 1 was assigned to forest while a value of 0 was allotted to non-forest areas (Fig 4).

**Fig 5:**
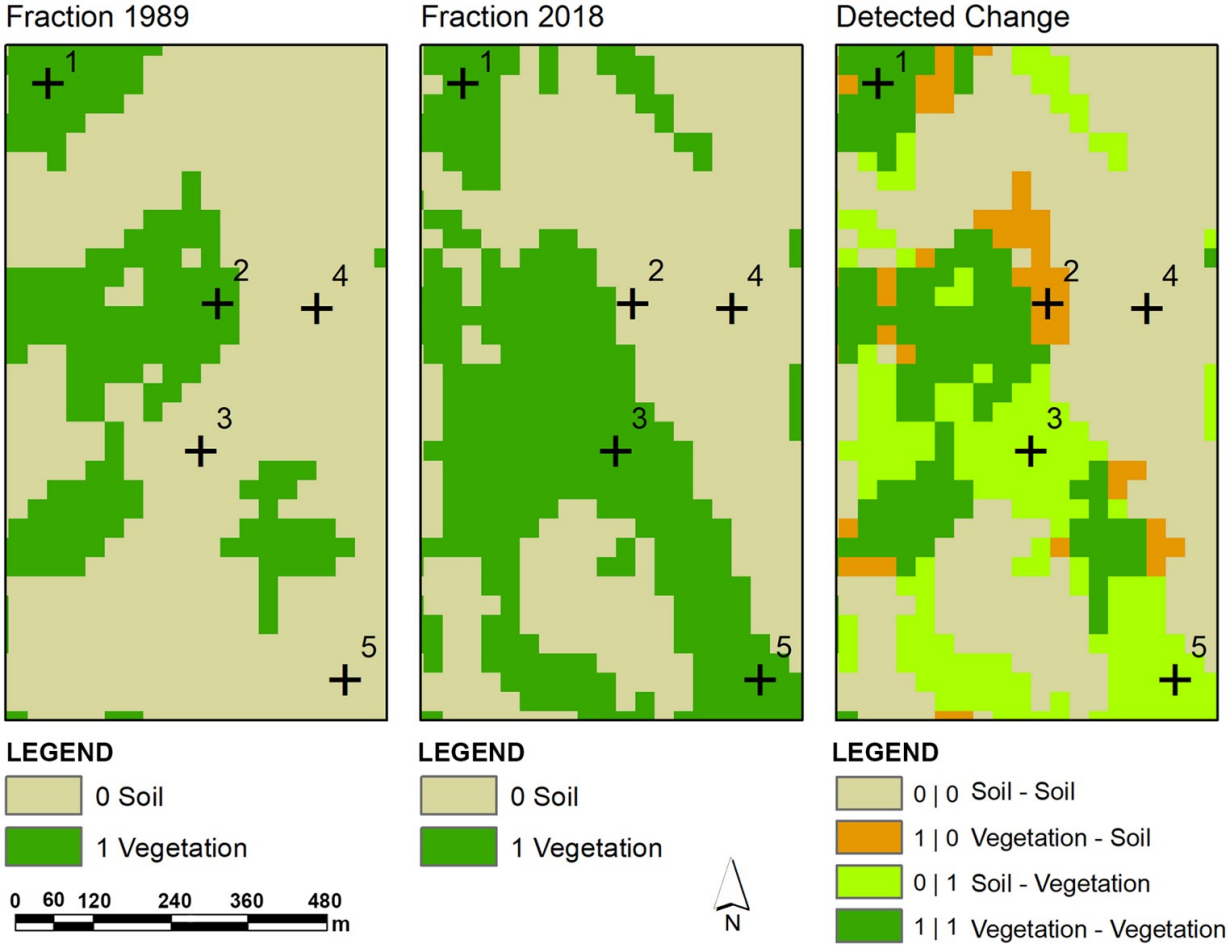
A subset of raster derived from cross tabulation analysis indicates class-class change (1989–2018).

Different change detection approaches were applied to produce multiple change maps. These change detection approaches and resultant maps helped better analyze landscape changes from different aspects (Fig 5).

A simple band combination applied to stacked images of TM-1989 and OLI-2018 revealed areas with abrupt changes in the vegetation cover. The stacked/composite image containing bands from both dates were used to produce qualitative information about changes in the landscape. The six TM bands (excluding TM band No. 6 i.e. Thermal band) were placed over top of the seven OLI bands spanned over visible and infrared portion of the spectrum. In the composite RGB stacked imaged, red color was assigned to band No. 13 (OLI SWIR 2), Green and Blue colors were designated to band no. 5 (TM SWIR 1) and band no. 8 (OLI Blue), respectively. The areas with increase in vegetation can be observed from distinct bright green color, while areas where vegetation has been removed and characterized by bare soil can be discerned as brighter shades of pink and magenta color. The analysis was performed in Arc GIS 10.5.

A cross tabulation of vegetation fractions derived from corresponding earlier and later date imagery (1989 and 2018) was performed to produce a composite change map. Earlier prepared binary rasters of vegetation fractions from both dates were compared through hard cross-classification approach to identify areas where change in the vegetation cover has occurred. The resultant composite change map enabled to categorize the type of change and provided additional information with respect to the change map produced from overlay of binary vegetation fraction rasters from earlier and later dates. The area and descriptive statistics were computed from quantifiable change maps as well as from fraction rasters to analyze land cover dynamics and associated biomass variations during the period.

### 2.5 Accuracy assessment

The classification results of latest Landsat 8 OLI scene (November, 2018) were assessed using the sample points collected during field survey. In order to analyze classification performance eighty points from forest and 60 points from non-forest cover classes were tested as referenced points to calculate producer’s accuracy, user’s accuracy, overall classification accuracy and Kappa statistics [40, 41, 42].

### 2.6 Assessment of forest carbon stock changes & CO_2_ emissions/sequestrations

**Fig 6:**
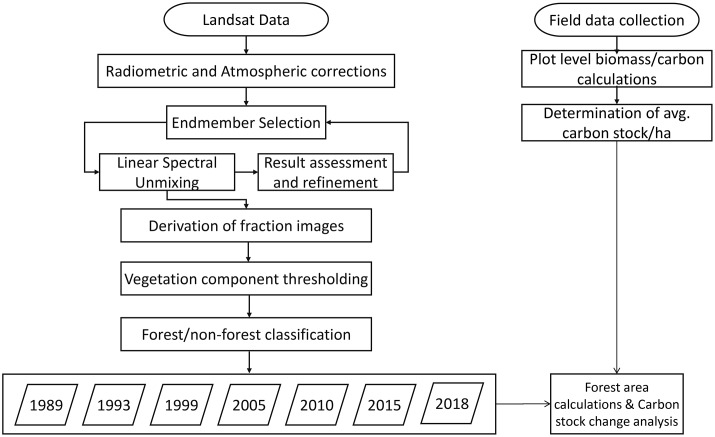
Process flow diagram.

The inventory approach [43] was adopted to measure the difference in carbon stocks averaged between two points in time. In order to convert biomass carbon to CO_2_, the tons of carbon were multiplied by a factor of 44/12 [44]. Forest area for each time was calculated from classified images and associated carbon stock was then estimated for all time periods accordingly. As the past forest inventory data (reflecting carbon stock density) was not available for the study area, the carbon stock difference between the intervals was calculated and according to the nature of stock change (positive or negative) corresponding CO_2_ emission /sequestration was estimated using the ACS value converted from inventory based AGB [45]. Flow chart of the process is illustrated in Fig 6.

## 3. Results

**Fig 7:**
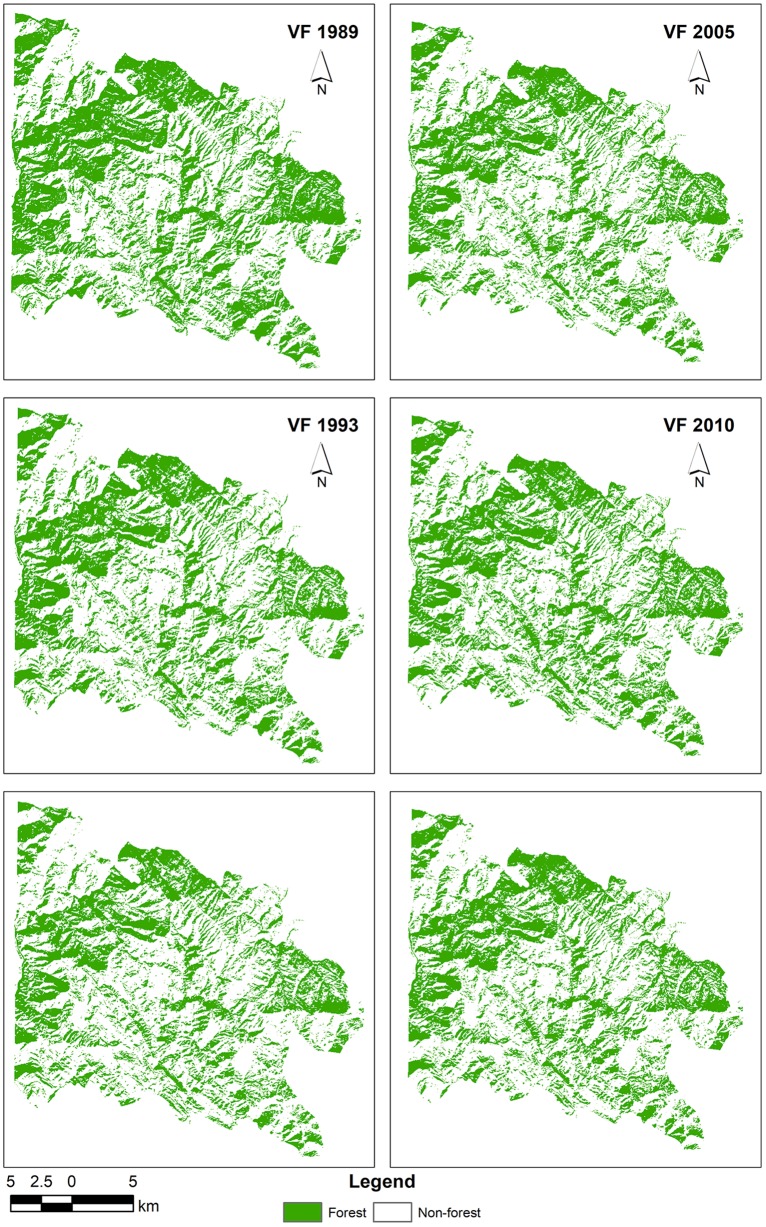
Classified vegetation fraction (VF) images (1989–2015).

**Fig 8:**
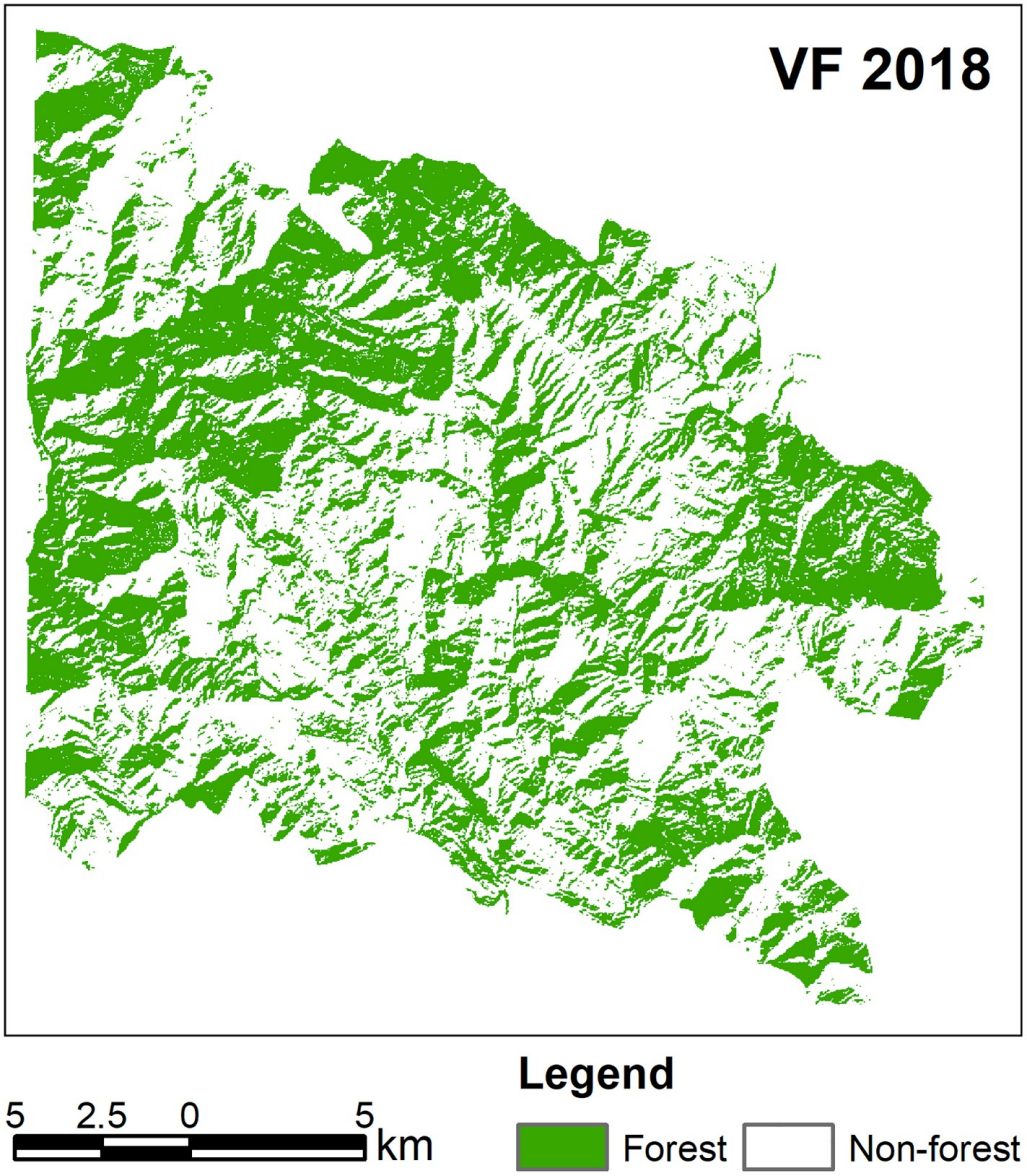
Classified vegetation fraction (VF) image (2018).

**Fig 9:**
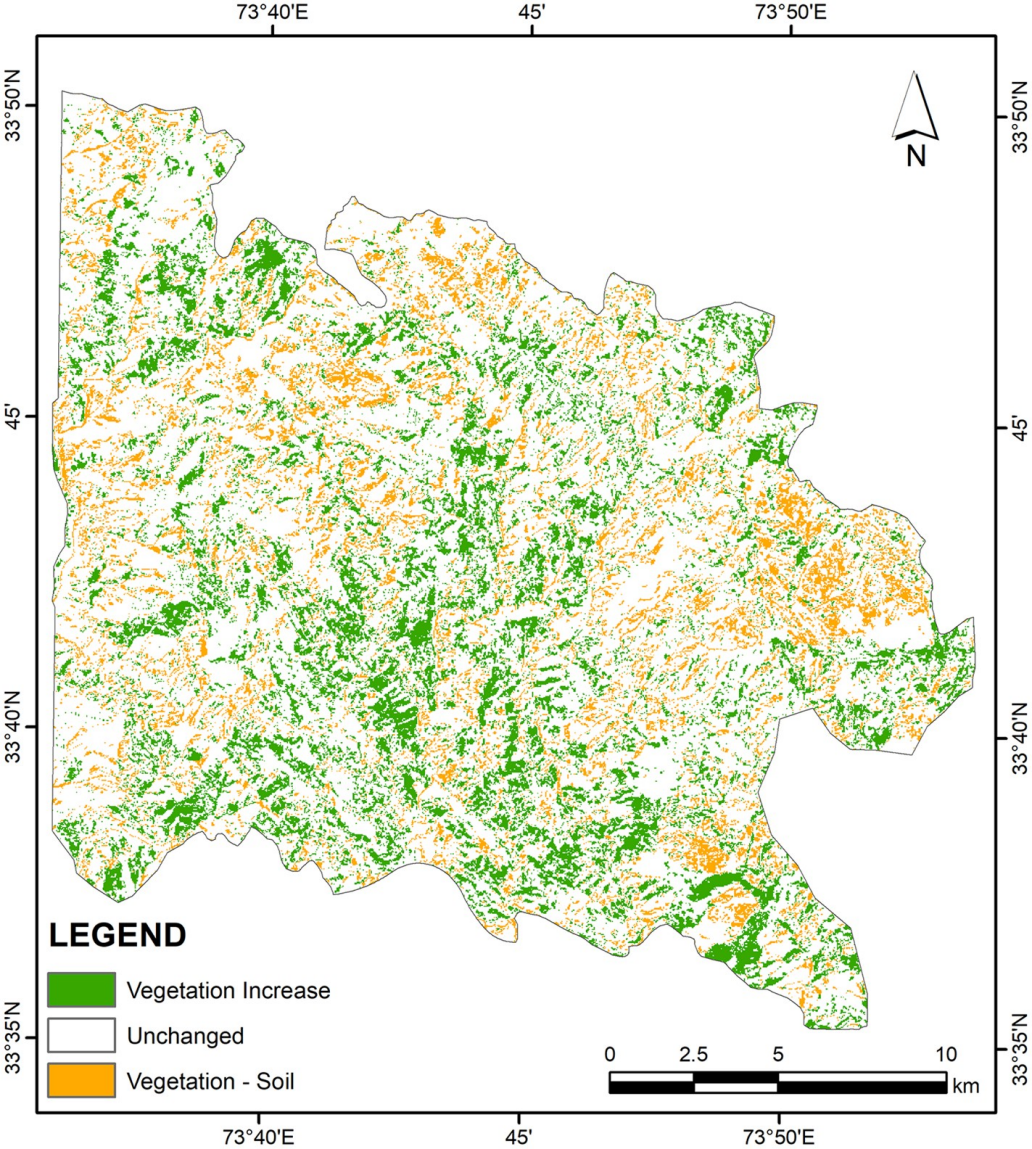
Vegetation cover change map produced using image overlay technique.

**Fig 10:**
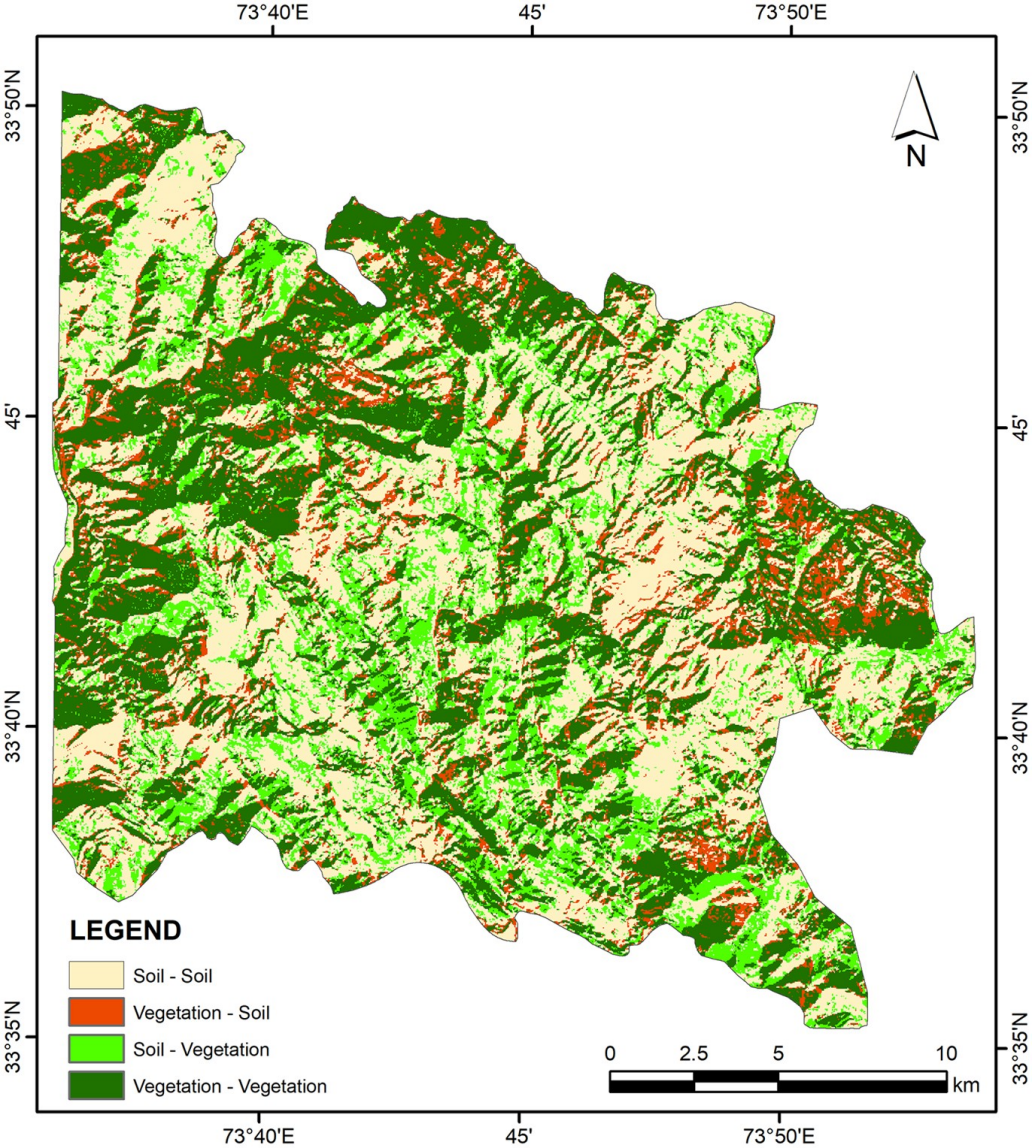
Class-class change map (1989–2018) produced using cross tabulation technique.

The estimated average above ground biomass (AAGB) was 0.145 Kt/ha with 0.072 Kt/ha corresponding carbon stock while average below ground biomass (ABGB) was 0.037 Kt/ha. The calculated producer’s accuracy for forest and non-forest classes was 97% and 95% respectively whereas 96% and 97% were the user’s accuracy values for these classes. Overall classification accuracy was 96% with Kappa coefficient value of 0.92 (S1 Table). Figs 7 and 8 exhibit classified vegetation fraction images while Fig 9 reflects increase or decrease in vegetation. Fig 10 illustrates change between classes over the study period (1989–2018).

**Fig 11:**
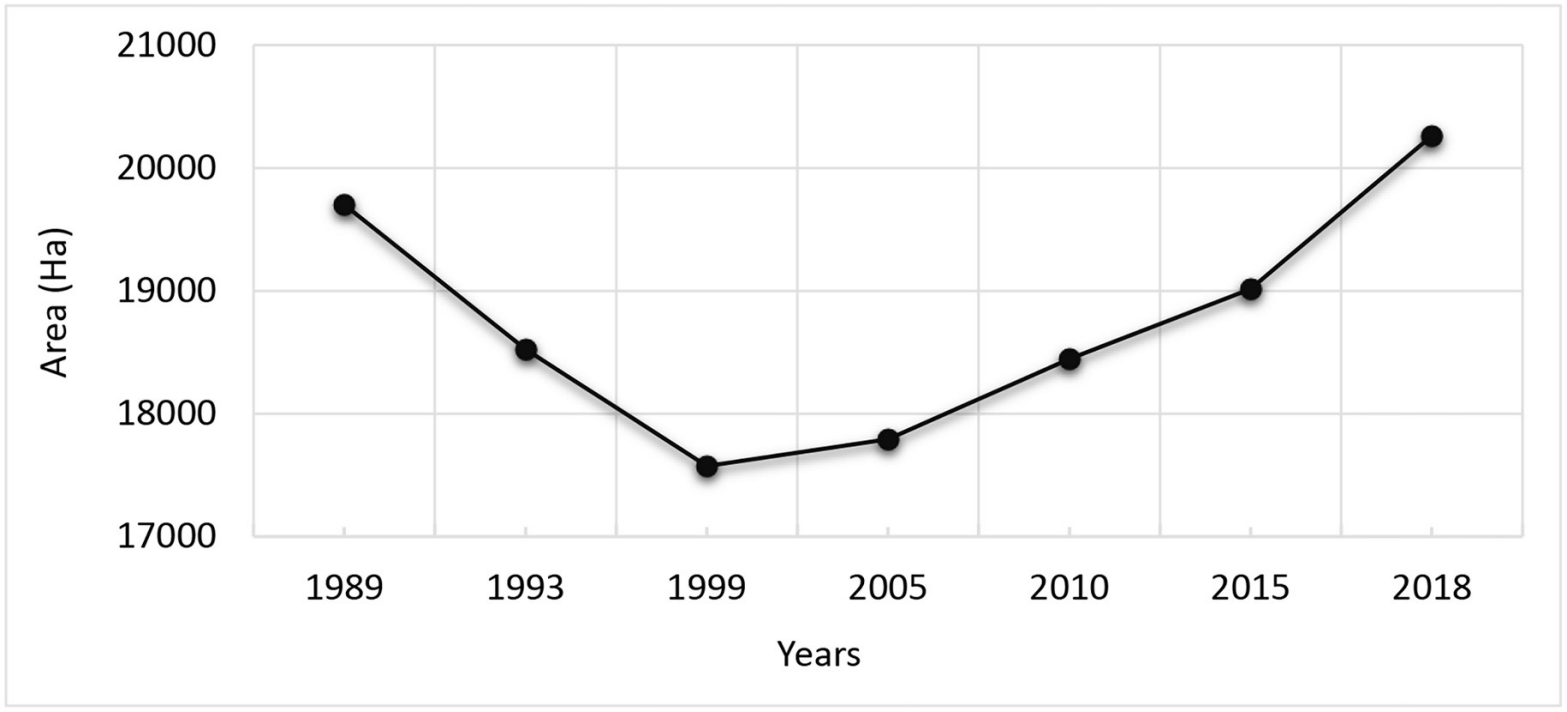
Trend of change in forest area from 1989–2018.

The year wise findings regarding forested area along with corresponding cover percentage and carbon stock is given in Table 2 while Fig 11 illustrates the changing trend of forest cover over the study period.

**Table 2:** Calculated forest area, forest cover % age and carbon stock.

Year	Forest Area (ha)	Cover (%age)	Total Carbon Stock (Kt)
**1989**	19701	41.82	1418.47
**1993**	18523	39.32	1333.56
**1999**	17574	37.31	1265.32
**2005**	17792	37.77	1281.02
**2010**	18447	39.16	1328.18
**2015**	19015	40.37	1369.08
**2018**	20262	43.01	1458.86

**Fig 12:**
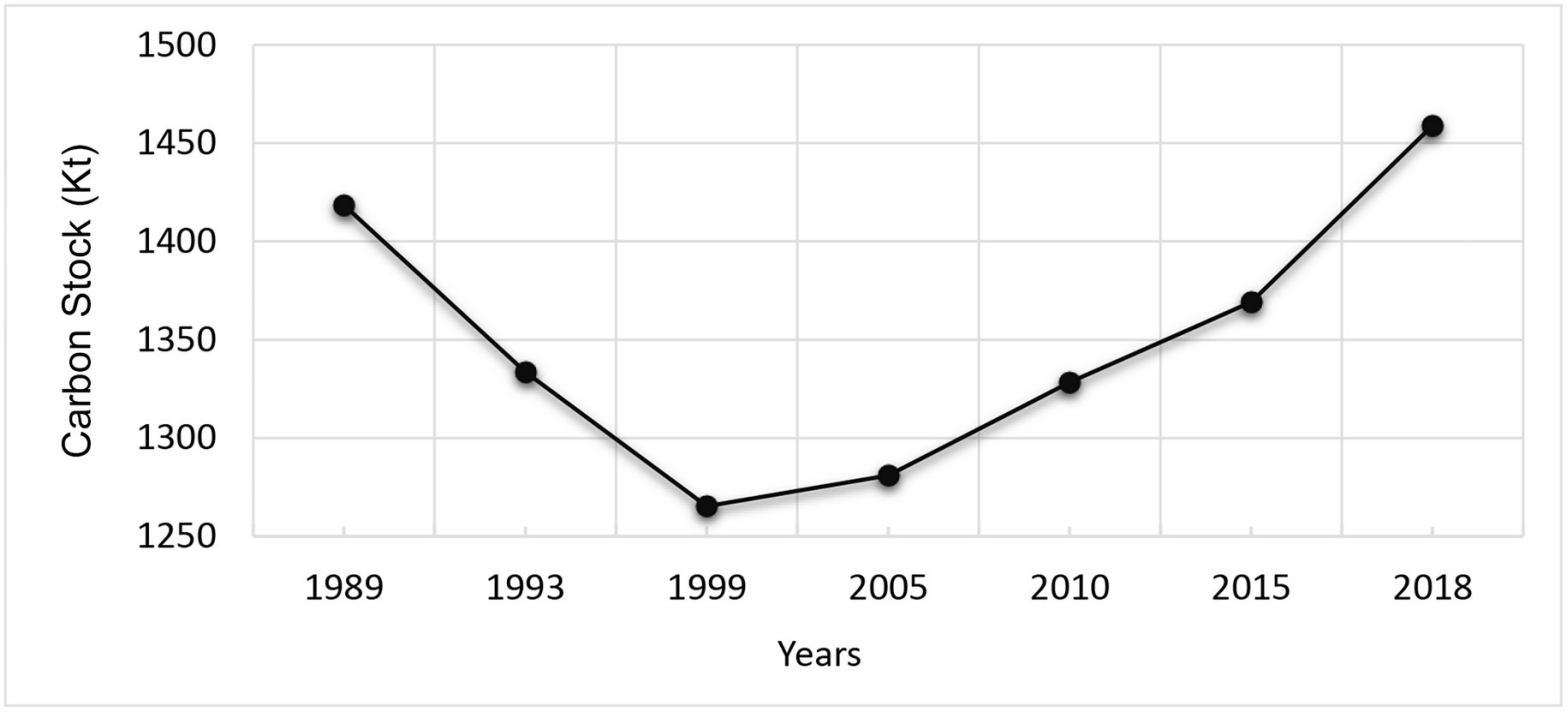
Changing trend in forest carbon stock from 1989–2018.

**Fig 13:**
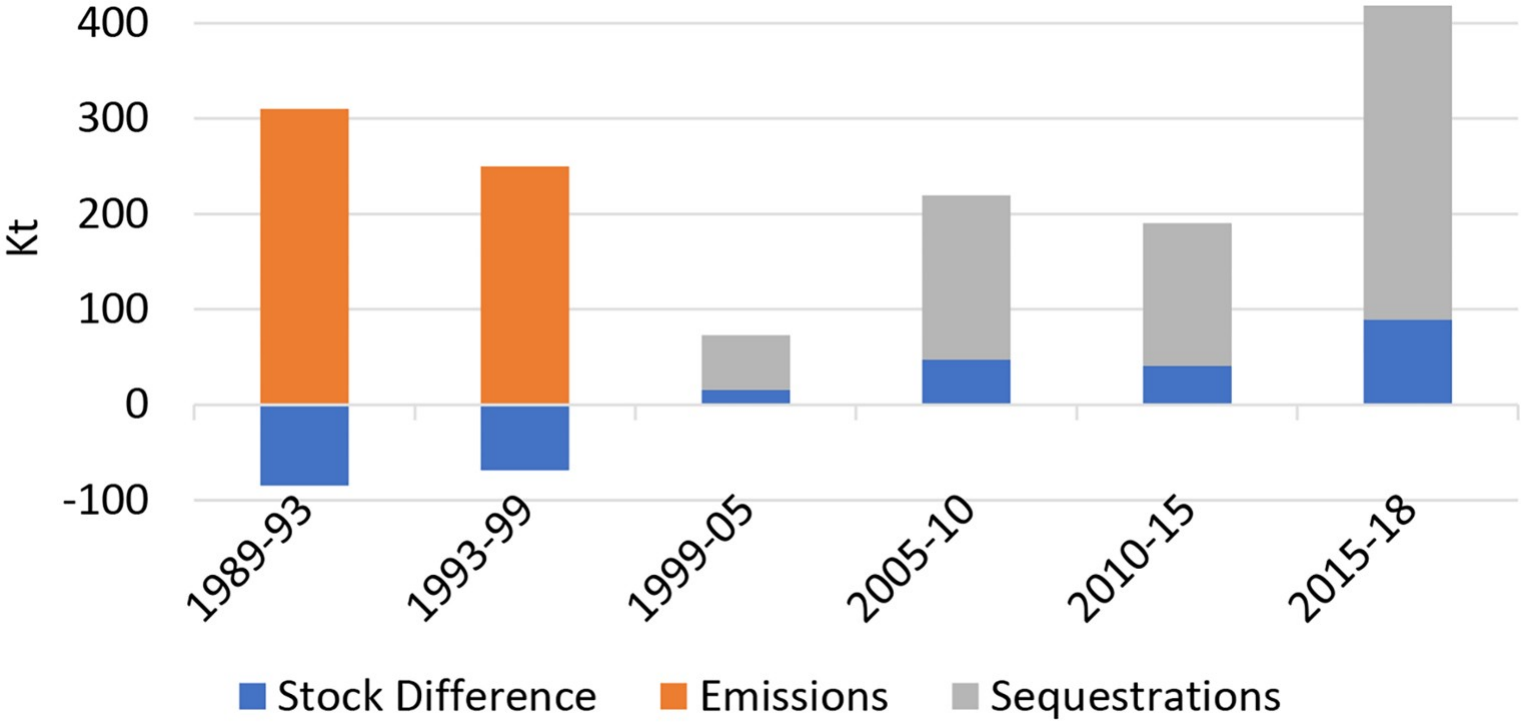
Changing trend in CO_2_ emissions & sequestrations from 1989–2018 with associated changes in forest carbon stock.

The periodic analysis revealed (Table 3) that major reduction in forest area (1178 ha) occurred during 1989–1999 imposing a negative change in forest cover from 41.82% to 39.32% followed by 949 ha during 1993–1999 with a decrease in forest cover from 39.32% to 37.31%. The minimum and maximum change in forest area over the study period is 218 ha and 1247 ha respectively with mean change of 802.5 ha (Table 4). For first two periods decrease in forest area resulted into decline in forest carbon stock by 84.81 Kt and 68.32 Kt with corresponding CO_2_ emissions by 310.40 Kt and 250.05 Kt, respectively (Table 3). However, a net gain of 2688 ha in forest area was observed during 1999–2018 which resulted into an increase in forest cover by 5.71% with 193.54 Kt increase in carbon stock leading to 708.34 Kt of corresponding CO_2_ sequestrations (Table 3). The changing trend in forest carbon stock resulting from change in forest area is depicted in Fig 12 while Fig 13 illustrates the changing trend in corresponding CO_2_ from emissions/sequestrations over the study period. Minimum and maximum change in forest carbon stock is represented by 15.69 Kt and 89.78 Kt with mean stock difference of 57.77 Kt (Table 4). Overall, the increase in forest area accounted for 561 ha resulting into 40.39 Kt increase in corresponding ACS during the study period. Net CO_2_ sequestrations (147.84 Kt) surpassed its emissions over a duration of about 25 years (Table 3).

**Table 3:** Carbon stock changes and corresponding emissions/sequestrations.

Time period	Change in Forest Area (ha)	Stock Difference (Kt)	Nature of Stock Change	Average.CO_2_ Emissions/Sequestrations (Kt)
**1989–1993**	1178	84.81	-ve	310.40
**1993–1999**	949	68.32	-ve	250.05
**1999–2005**	218	15.69	+ve	57.42
**2005–2010**	655	47.16	+ve	172.60
**2010–2015**	568	40.89	+ve	149.65
**2015–2018**	1247	89.78	+ve	328.59

**Table 4:** Descriptive statistics min, max, range, mean, Std. Error and Std. deviation of change in forest area and forest carbon stock.

	N	Range	Minimum	Maximum	Mean		Std. Deviation
					Statistic	Std. Error	
Change in Forest Area	6	1029.00	218.00	1247.00	802.5	161.08	394.57053
Stock Difference	6	74.09	15.69	89.78	57.7750	11.59790	28.40893
Valid N (list wise)	6						

## 4. Discussion

This study provides a comprehensive assessment of forest land cover and biomass carbon stock changes in a subtropical pine forest in AJK. Our estimation for overall average biomass for both components (AGB + BGB) resulted into 0.182 Kt/ha having 0.091 Kt/ha of carbon, over the study period. These results are in line with some studies conducted in close vicinity or same region. For example, Shaheen et al. [46] have reported an average biomass value of 0.192 Kt/ha from subtropical forest in Kashmir Himalaya. Similarly, biomass values of 0.237 Kt/ha and 0.186 Kt/ha have been reported by Nizami [47] for Ghoragali and Lehtrar Subtropical Pine *(Pinus roxburghii*) forests from Punjab, Pakistan, respectively. The forest condition / health may also have an impact on biomass outcome. For example, Jina et al. [48] have reported the carbon stock values ranging from 0.081–0.115 Kt/ha and 0.018–0.034 Kt/ha for non-degraded and degraded Chir pine (*Pinus roxburghii*) forest from Kuman Central Himalaya, respectively. However, it is well documented that forest carbon stock values are generally site specific and depend on geographic location, type of flora and age of tree stand [49, 50]. For example, in a study of similar nature conducted in sub-tropical to temperate zones of Garhwal Himalaya, Sharma et al. [51] have estimated significantly varying biomass stock values of 0.159 ± 0.016 Kt/ha and 0.298 ± 0.056 Kt/ha for Siwalik Chir Pine (*Pinus roxburghii*) and Himalayan Chir Pine (*Pinus roxburghii*) respectively.

**Fig 14:**
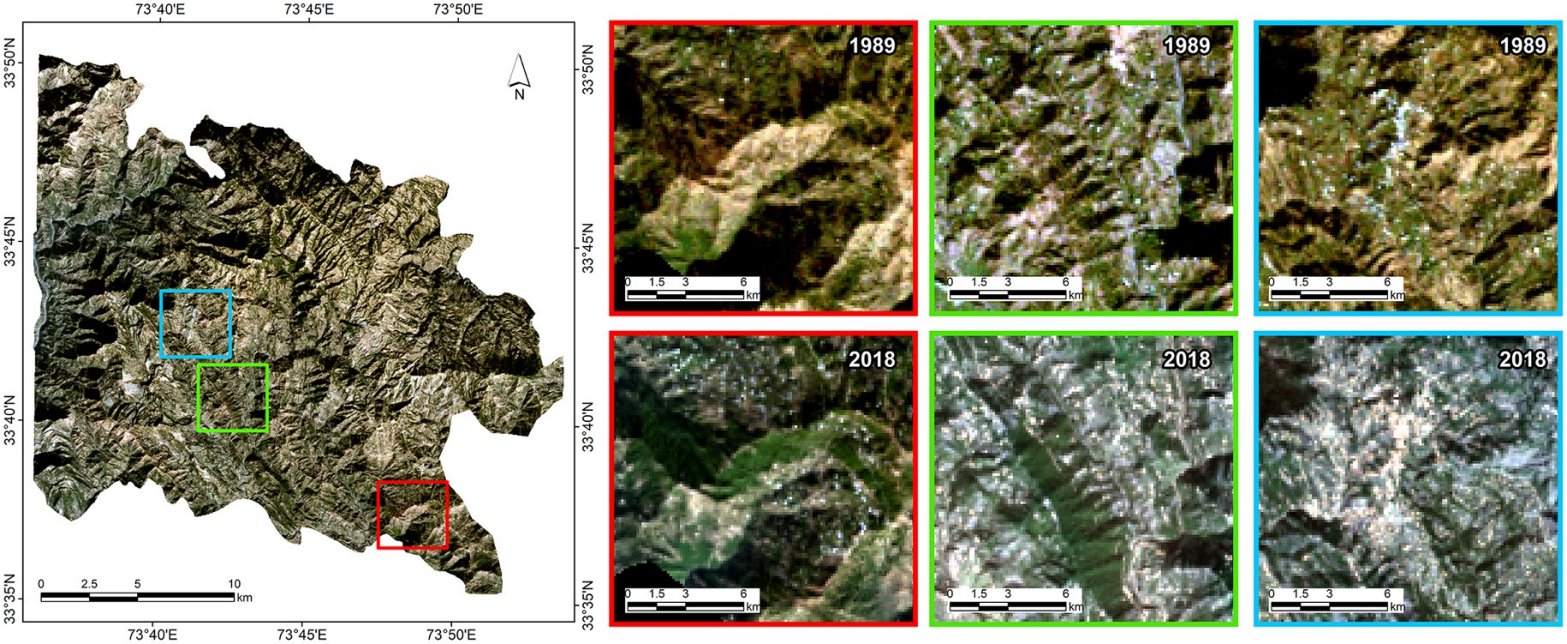
Areas of prominent change in forest cover (1989 & 2018) with reference to their grid position.

**Fig 15:**
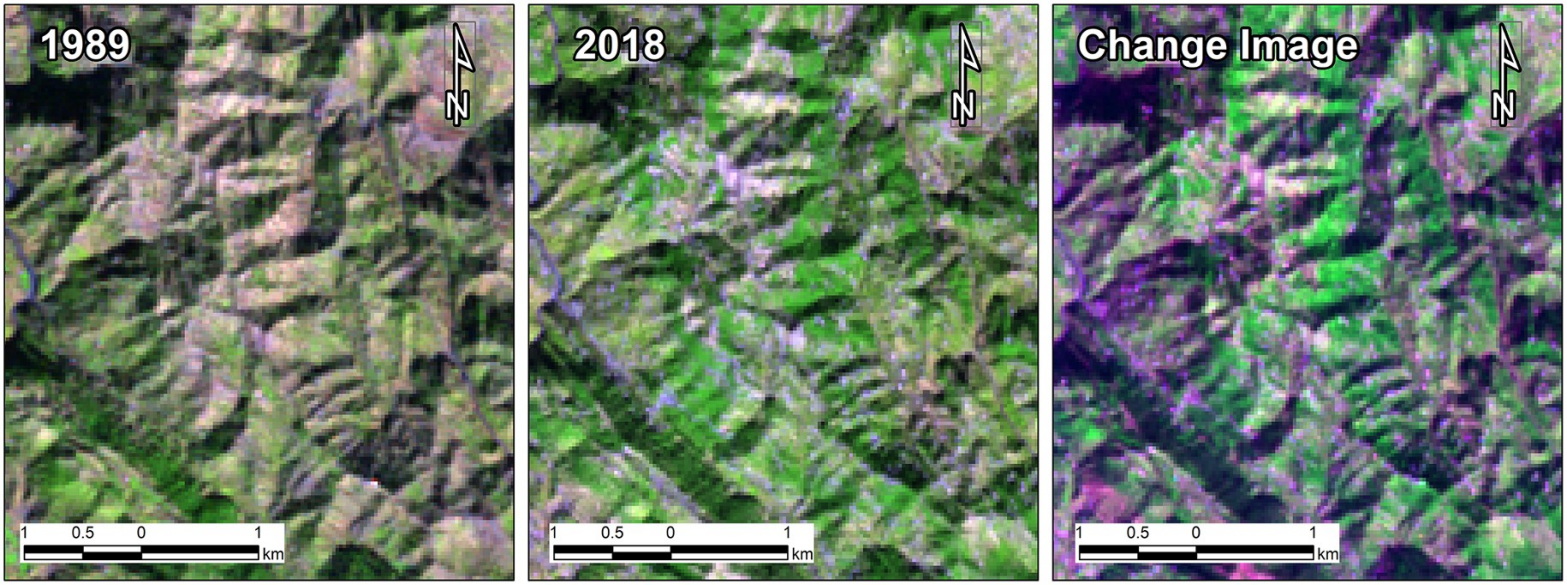
Composite spectral change image highlights the areas of vegetation gain, loss and no change during the period 1989–2018.

Overall, there has been a net increase of 561 ha in forest area with increase of 40.39 Kt in corresponding biomass carbon stock. The sharp reduction in forest area by 2127 ha (11%) during 1989–1999 may be attributed to legalized commercial exploitation of mature trees by the GoAJK to earn revenue coupled with huge dependency of rural communities for timber and fuelwood to meet their needs for construction and heat /cooking at household level. The increase in population also led to increased built up areas in town and villages while forest land encroachment may had a negative impact on forest area and land cover. The land cover changes have been reported to be influenced by the increase in human population [52] such as in Ethiopia, population growth has been reported a dominant cause of land cover change in comparison to other factors [53]. However, regain in forest cover during 1999–2018 can be attributed to a complete ban imposed by the GoAJK on cutting of green trees and launching of reforestation and social forestry programmes/projects in the State. During the study period, State forest department established plantations on 20,442 ha under different development projects with significant success. This not only enhanced protection of existing forests /regeneration but planting on communal and private lands was encouraged through community organizations and mass awareness. Moreover, from response to a questionnaire distributed among the local dwellers as a part of social survey, it was evident that many other social and economic factors have played a notable role in restoring deforestation losses and improving the forest cover. It indicates that family income has increased during last two decades and it has resulted into uplift of living standards. It was revealed that migration trend from villages to big cities like Rawalpindi/Islamabad in seek of quality health and education increased over last two decades and it helped reduce burden on fuel wood and timber consumption. Majority of the respondents were of the view that provision of electricity lead to reduced usage of fuel wood and access to fuel wood and timber alternatives became very convenient with the extension of link roads network. It is believed by 68% of respondents that lowering trend in livestock rearing that declined during last two decades has contributed significantly towards forest cover improvement and that growing trees on private lands in order to meet fuel wood and fodder requirements has increased too over this period. The gain in forest cover substantiates the success of these projects which have been able to convince the local forest dependent communities to promote social/farm forestry and to switch over to alternate sources or substitutes of fuel wood and timber. The areas of vegetation gain and loss can be seen highlighted in geo-linked Landsat images of 1989 and 2018 (Fig 14) and a spectral change image produced through a band composite of Landsat images (Landsat TM -1989 and Landsat OLI -2018) enables to view the places of vegetation gain, loss and no change (Fig 15) during the study period.

It was encouraging to find that this also follows the recently revealed global trend through the Global Forest Resources Assessment 2015 that during 1990–2015 deforestation has slowed and afforestation has increased globally [54]. Moreover, it is recognized now that the involvement of local stakeholders towards owning and managing forests is increasing [55] and the importance of forest management as a source for combating climate change is also realized worldwide [56].

## 5. Conclusion

Remote sensing is a dynamic technological advancement which provides fast, economical and reliable information necessary for spatiotemporal assessment, planning and management of complex natural resources, especially forests. Our endeavor to estimate intervallic forest cover and carbon stock changes for a Subtropical Pine Forest in AJK confirmed an average biomass of 0.181 Kt/ha with corresponding carbon value of 0.091 Kt/ha. Our assessment shows a drastic reduction in forest area by 2127 ha (11%) over a period of one decade (1989–1999) attributed to commercial exploitation of forests at State level, urban built up, heavy dependency of rural communities on forests for timber and fuelwood along with increase in population and forest land encroachment. However, a net gain of 2688 ha in forest area was observed during 1999–2018 attributed to ban on cutting of green trees, mass awareness and planting of 20,442 ha under different reforestation and social forestry programmes/projects. We recommend the State forest department to incorporate information regarding carbon stock and CO_2_ emissions/sequestrations in future inventories through assessment using remote sensing especially Landsat data archive. This information can prove to be very useful while preparing, implementing and monitoring the forest resources development projects intended to protect and conserve forests, check encroachments on forest land and enhance forest carbon stocks with ultimate aim of reducing emissions and increasing sequestrations. We also stress to further promote reforestation and social forestry projects and minimize communities’ dependency on forests through increase in provision of wood substitutes especially for construction and fuelwood.

## Supporting information

S1 Fig Photographs of field activities. (DOCX)

S2 Fig Photographs of instruments used for field inventory. (DOCX)

S1 File Coordinates of field inventory plots. (PDF)

S1 Table Error matrix for Landsat OLI 2018 image classification. (DOCX)

## References

[1] Keenan RJ, Reams GA, Achard F, de Freitas JV, Grainger A, Lindquist E. Dynamics of global forest area: Results from the FAO Global Forest Resources Assessment 2015. For Ecol Manage. 2015;352:9–20. 10.1016/j.foreco.2015.06.014

[2] Kumar KK, Nagai M, Witayangkurn A, Kritiyutanant K, Nakamura S. Above Ground Biomass Assessment from Combined Optical and SAR Remote Sensing Data in Surat Thani Province, Thailand. Journal of Geographic Information System. 2016;8:506 10.4236/jgis.2016.84042

[3] Houghton R, Hall F, Goetz SJ. Importance of biomass in the global carbon cycle. J Geophys Res Biogeosciences. 2009;114 10.1029/2009JG000935

[4] Gibbs HK, Brown S, Niles JO, Foley JA. Monitoring and estimating tropical forest carbon stocks: making REDD a reality. Environ Res Lett. 2007;2:045023 10.1088/1748-9326/2/4/045023

[5] Stern N, Stern NH. The economics of climate change: the Stern review: cambridge University press; 2007.

[6] FAO. Global forest resource assessment 2005: progress towards Sustainable forest management. FAO forestry Paper 147 Rome: FAO; 2006. 2005

[7] Dibaba A, Soromessa T, Workineh B. Carbon stock of the various carbon pools in Gerba-Dima moist Afromontane forest, South-western Ethiopia. Carbon balance and management. 2019;14:1 10.1186/s13021-019-0116-x 30712188 PMC6446976

[8] Satish K, Saranya K, Reddy CS, Krishna PH, Jha C, Rao PP. Geospatial assessment and monitoring of historical forest cover changes (1920–2012) in Nilgiri Biosphere Reserve, Western Ghats, India. Environ Monit Assess. 2014;186:8125–8140. 10.1007/s10661-014-3991-3 25117494

[9] Ningthoujam R, Tansey K, Balzter H, Morrison K, Johnson S, Gerard F, et al Mapping forest cover and forest cover change with airborne S-band radar. Remote Sens. 2016;8:577 10.3390/rs8070577

[10] Potapov PV, Turubanova SA, Hansen MC, Adusei B, Broich M, Altstatt A, et al Quantifying forest cover loss in Democratic Republic of the Congo, 2000–2010, with Landsat ETM+ data. Remote Sens Environ. 2012;122:106–116. 10.1016/j.rse.2011.08.027

[11] Furby S. Land cover change: specification for remote sensing analysis. 2002

[12] Maliqi E, Penev P. Monitoring of vegetation change by using RS and GIS techniques in Mitrovica, Kosovo. Journal of Cartography and Geographic Information Systems. 2018;1:1–13. 10.23977/jcgis.2018.11001

[13] Huang C, Song K, Kim S, Townshend JR, Davis P, Masek JG, et al Use of a dark object concept and support vector machines to automate forest cover change analysis. Remote Sens Environ. 2008;112:970–985. 10.1016/j.rse.2007.07.023

[14] Souza CM Jr, Siqueira JV, Sales MH, Fonseca AV, Ribeiro JG, Numata I, et al Ten-year Landsat classification of deforestation and forest degradation in the Brazilian Amazon. Remote Sens. 2013;5:5493–5513. 10.3390/rs5115493

[15] Souza C, Firestone L, Silva LM, Roberts D. Mapping forest degradation in the Eastern Amazon from SPOT 4 through spectral mixture models. Remote Sens Environ. 2003;87:494–506. 10.1016/j.rse.2002.08.002

[16] Renó VF, Novo EM, Suemitsu C, Renno CD, Silva TS. Assessment of deforestation in the Lower Amazon floodplain using historical Landsat MSS/TM imagery. Remote Sens Environ. 2011;115:3446–3456. 10.1016/j.rse.2011.08.008

[17] Phua M-H, Tsuyuki S, Furuya N, Lee JS. Detecting deforestation with a spectral change detection approach using multitemporal Landsat data: A case study of Kinabalu Park, Sabah, Malaysia. Journal of Environmental Management. 2008;88:784–795. 10.1016/j.jenvman.2007.04.011 17629393

[18] Liu T, Yang X. Mapping vegetation in an urban area with stratified classification and multiple endmember spectral mixture analysis. Remote Sens Environ. 2013;133:251–264. 10.1016/j.rse.2013.02.020

[19] Karimi N, Golian S, Karimi D. Monitoring deforestation in Iran, Jangal-Abr Forest using multi-temporal satellite images and spectral mixture analysis method. Arab J Geosci. 2016;9:214 10.1007/s12517-015-2250-4

[20] Deng Y, Wu C. Development of a class-based multiple endmember spectral mixture analysis (C-MESMA) approach for analyzing urban environments. Remote Sens. 2016;8:349 10.3390/rs8040349

[21] Thenkabail PS, Biradar CM, Noojipady P, Dheeravath V, Li Y, Velpuri M, et al Global irrigated area map (GIAM), derived from remote sensing, for the end of the last millennium. Int J Remote Sens. 2009;30:3679–3733. 10.1080/01431160802698919

[22] Gong P, Wang J, Yu L, Zhao Y, Zhao Y, Liang L, et al Finer resolution observation and monitoring of global land cover: First mapping results with Landsat TM and ETM+ data. Int J Remote Sens. 2013;34:2607–2654. 10.1080/01431161.2012.748992

[23] Townshend JR, Masek JG, Huang C, Vermote EF, Gao F, Channan S, et al Global characterization and monitoring of forest cover using Landsat data: opportunities and challenges. Int J Digit Earth. 2012;5:373–397. 10.1080/17538947.2012.713190

[24] Jia K, Li Q, Tian Y, Wu B, Zhang F, Meng J. Crop classification using multi-configuration SAR data in the North China Plain. Int J Remote Sens. 2012;33:170–183. 10.1080/01431161.2011.587844

[25] Nordberg ML, Evertson J. Vegetation index differencing and linear regression for change detection in a Swedish mountain range using Landsat TM^®^ and ETM+^®^ imagery. Land Degradation & Development. 2005;16:139–149. 10.1002/ldr.660

[26] Langley SK, Cheshire HM, Humes KS. A comparison of single date and multitemporal satellite image classifications in a semi-arid grassland. J Arid Environ. 2001;49:401–411. 10.1006/jare.2000.0771

[27] Tomppo E, Gschwantner T, Lawrence M, McRoberts RE, Gabler K, Schadauer K, et al. National forest inventories. Pathways for Common Reporting European Science Foundation. 2010:541–553

[28] Xie Y, Sha Z, Yu M. Remote sensing imagery in vegetation mapping: a review. J Plant Ecol. 2008;1:9–23. 10.1093/jpe/rtm005

[29] Siddiqui K. Asia-Pacific Forestry Sector Outlook Study. Country Report—Pakistan. Working paper no: APFSOS/WP/11 Food and Agriculture Organization of the United Nations. 1997

[30] Pearson T, Walker S, Brown S. Sourcebook for land use, land-use change and forestry projects. Biocarbon Fund and Winrock International. 2005.

[31] Malhi Y, Baker TR, Phillips OL, Almeida S, Alvarez E, Arroyo L, et al The above‐ground coarse wood productivity of 104 Neotropical forest plots. Glob Chang Biol. 2004;10:563–591. 10.1111/j.1529-8817.2003.00778.x

[32] Basuki T, Van Laake P, Skidmore A, Hussin Y. Allometric equations for estimating the above-ground biomass in tropical lowland Dipterocarp forests. For Ecol Manage. 2009;257:1684–1694. 10.1016/j.foreco.2009.01.027

[33] Quintano C, Fernández-Manso A, Shimabukuro YE, Pereira G. Spectral unmixing. Int J Remote Sens. 2012;33:5307–5340. 10.1080/01431161.2012.661095

[34] Smith MO, Ustin SL, Adams JB, Gillespie AR. Vegetation in deserts: I. A regional measure of abundance from multispectral images. Remote Sens Environ. 1990;31:1–26. 10.1016/0034-4257(90)90074-V

[35] Lu D, Moran E, Batistella M. Linear mixture model applied to Amazonian vegetation classification. Remote Sens Environ. 2003;87:456–469. 10.1016/j.rse.2002.06.001

[36] Adams JB, Sabol DE, Kapos V, Almeida Filho R, Roberts DA, Smith MO, et al Classification of multispectral images based on fractions of endmembers: Application to land-cover change in the Brazilian Amazon. Remote Sens Environ. 1995;52:137–154. 10.1016/0034-4257(94)00098-8

[37] Lunetta RS, and Christopher Elvidge. Remote Sensing Change Detection: Environmental Monitoring Methods and Applications: Chelsea, Mich: Ann Arbor Press; 1998.

[38] Collado AD, Chuvieco E, Camarasa A. Satellite remote sensing analysis to monitor desertification processes in the crop-rangeland boundary of Argentina. J Arid Environ. 2002;52:121–133. 10.1006/jare.2001.0980

[39] Salih AA, Ganawa E-T, Elmahl AA. Spectral mixture analysis (SMA) and change vector analysis (CVA) methods for monitoring and mapping land degradation/desertification in arid and semiarid areas (Sudan), using Landsat imagery. Egypt J Remote Sens Sp Sci. 2017;20:S21–S29. 10.1016/j.ejrs.2016.12.008

[40] Foody GM. Ground reference data error and the mis-estimation of the area of land cover change as a function of its abundance. Remote Sens Lett. 2013;4:783–792. 10.1080/2150704X.2013.798708

[41] Foody GM. Classification accuracy comparison: hypothesis tests and the use of confidence intervals in evaluations of difference, equivalence and non-inferiority. Remote Sens Environ. 2009;113:1658–1663. 10.1016/j.rse.2009.03.014

[42] Congalton RG, Green K. Assessing the accuracy of remotely sensed data: principles and practices: CRC press; 2008.

[43] McRoberts RE, Næsset E, Gobakken T. Comparing the stock-change and gain–loss approaches for estimating forest carbon emissions for the aboveground biomass pool. Canadian Journal of Forest Research. 2018;48:1535–1542. 10.1139/cjfr-2018-0295

[44] Watson C. Forest carbon accounting: overview and principles. For carbon Account Overv Princ. 2009

[45] Munawar S, Khokhar MF, Atif S. Reducing emissions from deforestation and forest degradation implementation in northern Pakistan. Int Biodeterior Biodegradation. 2015;102:316–323. 10.1016/j.ibiod.2015.02.027

[46] Shaheen H, Khan RWA, Hussain K, Ullah TS, Nasir M, Mehmood A. Carbon stocks assessment in subtropical forest types of Kashmir Himalayas. Pak J Bot. 2016;48:2351–2357

[47] Nizami SM. The inventory of the carbon stocks in sub tropical forests of Pakistan for reporting under Kyoto Protocol. Journal of Forestry Research. 2012;23:377–384. 10.1007/s11676-012-0273-1

[48] Jina B, Sah P, Bhatt M, Rawat Y. Estimating carbon sequestration rates and total carbon stockpile in degraded and non-degraded sites of Oak and Pine forest of Kumaun Central Himalaya. Ecoprint: An International Journal of Ecology. 2009;15:75–81. 10.3126/eco.v15i0.1946

[49] van Noordwijk M, Cerri C, Woomer PL, Nugroho K, Bernoux M. Soil carbon dynamics in the humid tropical forest zone. Geoderma. 1997;79:187–225. 10.1016/S0016-7061(97)00042-6

[50] Harmon ME, Hua C. Coarse woody debris dynamics in two old-growth ecosystems. BioScience. 1991;41:604–610. 10.2307/1311697

[51] Sharma CM, Baduni NP, Gairola S, Ghildiyal SK, Suyal S. Tree diversity and carbon stocks of some major forest types of Garhwal Himalaya, India. For Ecol Manage. 2010;260:2170–2179. 10.1016/j.foreco.2010.09.014

[52] Geist HJ, Lambin EF. Proximate causes and underlying driving forces of tropical deforestation: Tropical forests are disappearing as the result of many pressures, both local and regional, acting in various combinations in different geographical locations. BioScience. 2002;52:143–150. 10.1641/0006-3568(2002)052[0143:PCAUDF]2.0.CO;2

[53] Turner BL, Meyer WB. Global land-use and land-cover change: an overview. Changes in land use and land cover: a global perspective. 1994;4

[54] Sloan S, Sayer JA. Forest Resources Assessment of 2015 shows positive global trends but forest loss and degradation persist in poor tropical countries. For Ecol Manage. 2015;352:134–145. 10.1016/j.foreco.2015.06.013

[55] MacDicken KG, Sola P, Hall JE, Sabogal C, Tadoum M, de Wasseige C. Global progress toward sustainable forest management. For Ecol Manage. 2015;352:47–56. 10.1016/j.foreco.2015.02.005

[56] Federici S, Tubiello FN, Salvatore M, Jacobs H, Schmidhuber J. New estimates of CO2 forest emissions and removals: 1990–2015. For Ecol Manage. 2015;352:89–98. 10.1016/j.foreco.2015.04.022

