# Supplementary figures and images for: Assessment of forest cover and carbon stock changes in sub-tropical pine forest of Azad Jammu & Kashmir (AJK), Pakistan using multi-temporal Landsat satellite data and field inventory
Source: PLoS One. 2020 Jan 23;15(1):e0226341. doi: 10.1371/journal.pone.0226341 (PMC6977729; doi:10.1371/journal.pone.0226341)

**Figure S1: Different Activities during field inventory**


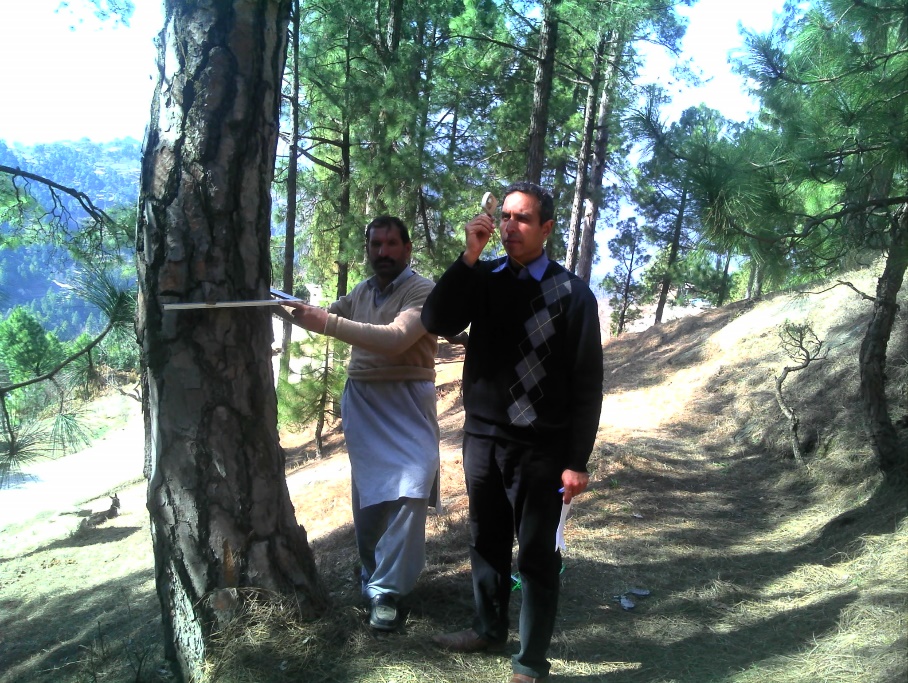

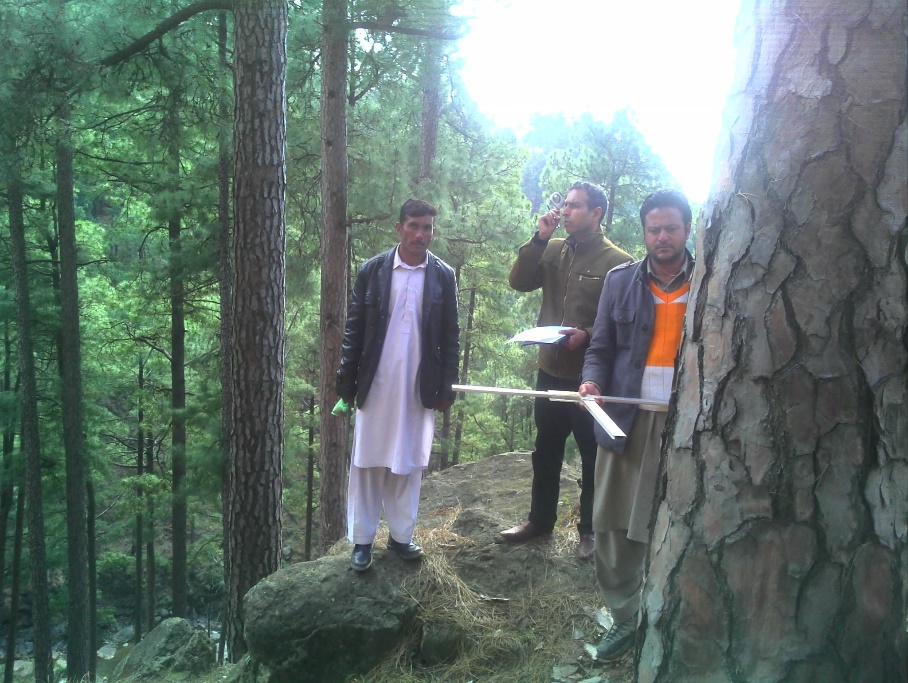

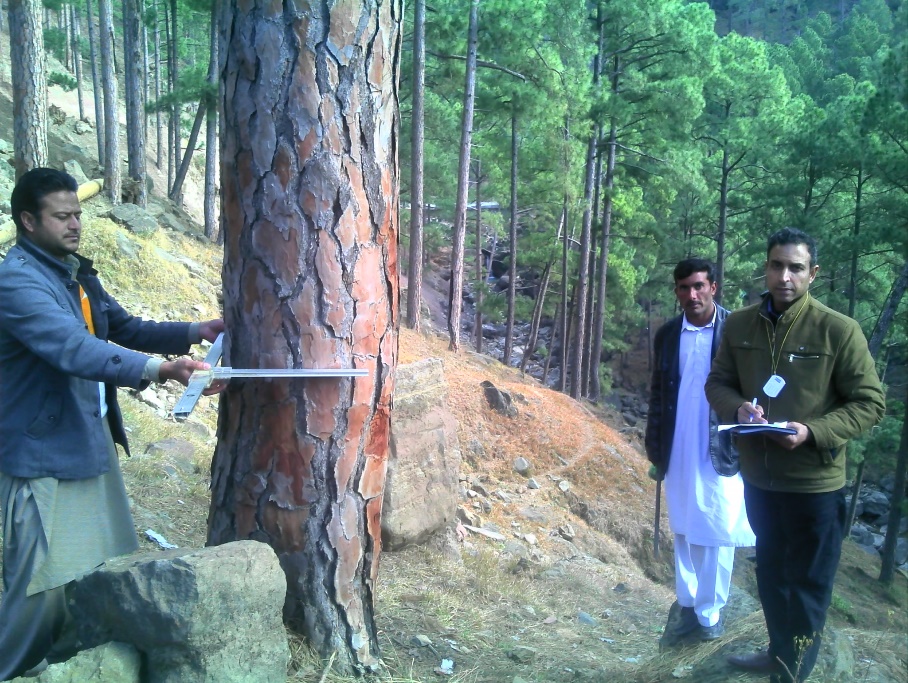

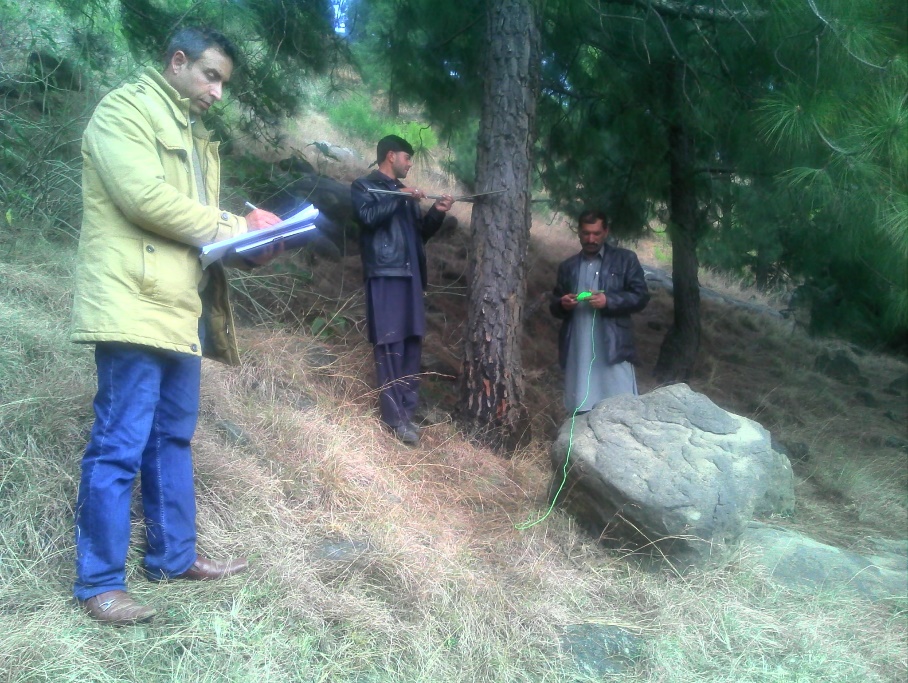

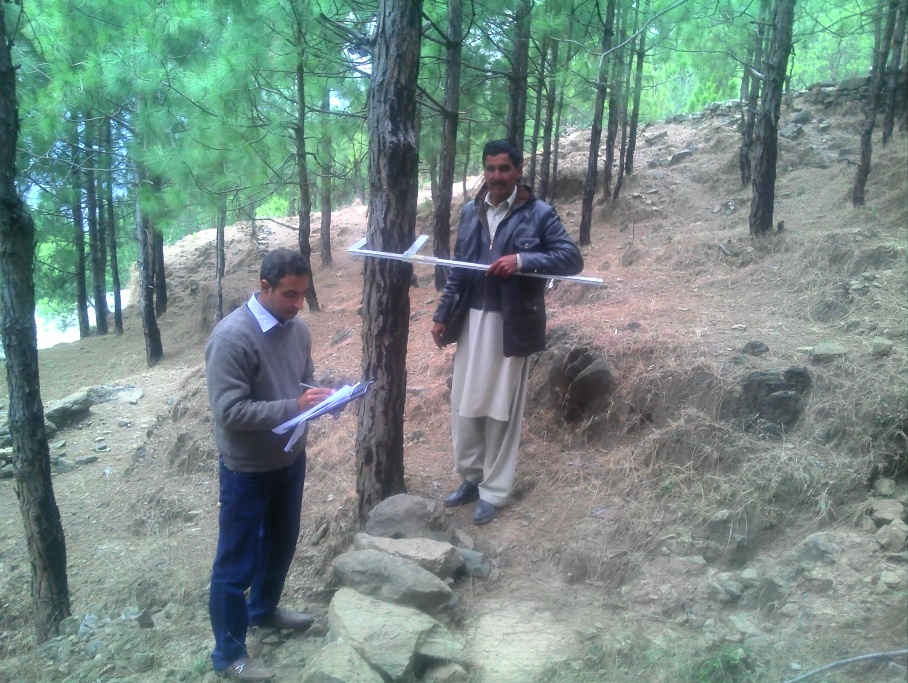

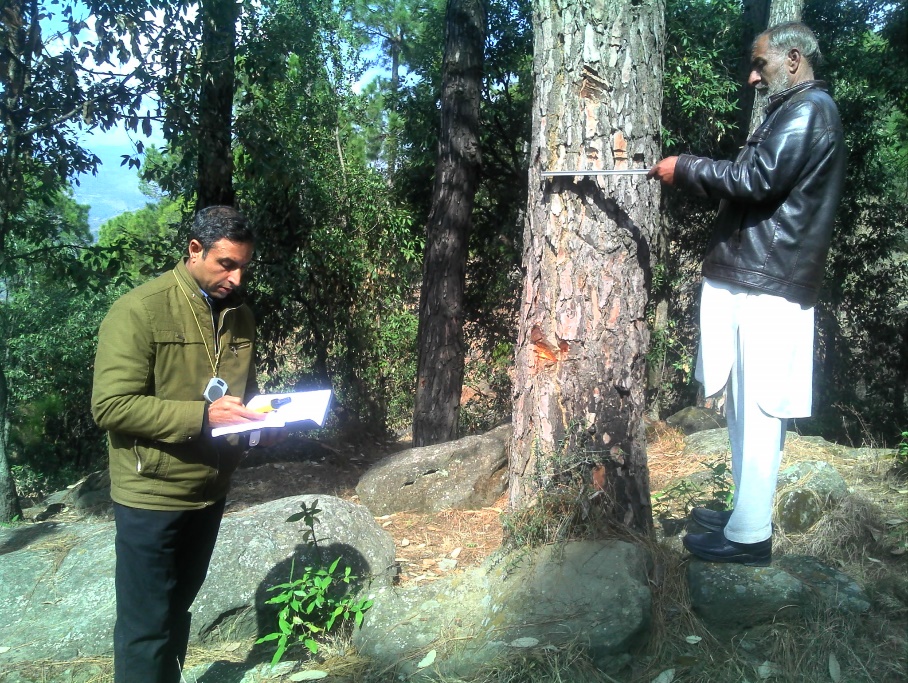

Supplement: S1 Fig — (DOCX) [file pone.0226341.s001.docx]
