# Supplementary material for: Assessment of forest cover and carbon stock changes in sub-tropical pine forest of Azad Jammu & Kashmir (AJK), Pakistan using multi-temporal Landsat satellite data and field inventory
Source: PLoS One. 2020 Jan 23;15(1):e0226341. doi: 10.1371/journal.pone.0226341 (PMC6977729; doi:10.1371/journal.pone.0226341)
Supplement: S2 Fig — (DOCX) [file pone.0226341.s002.docx]

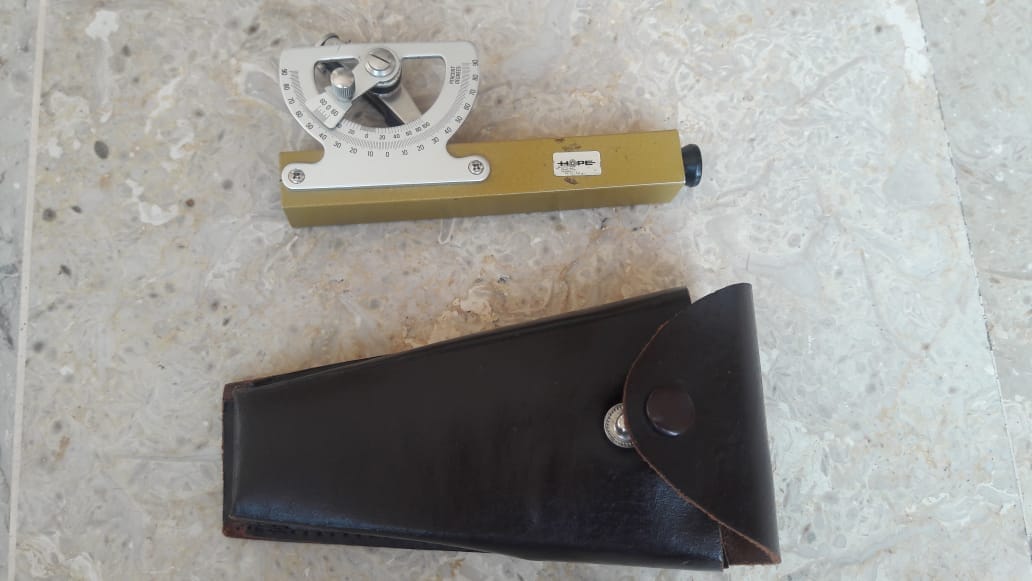

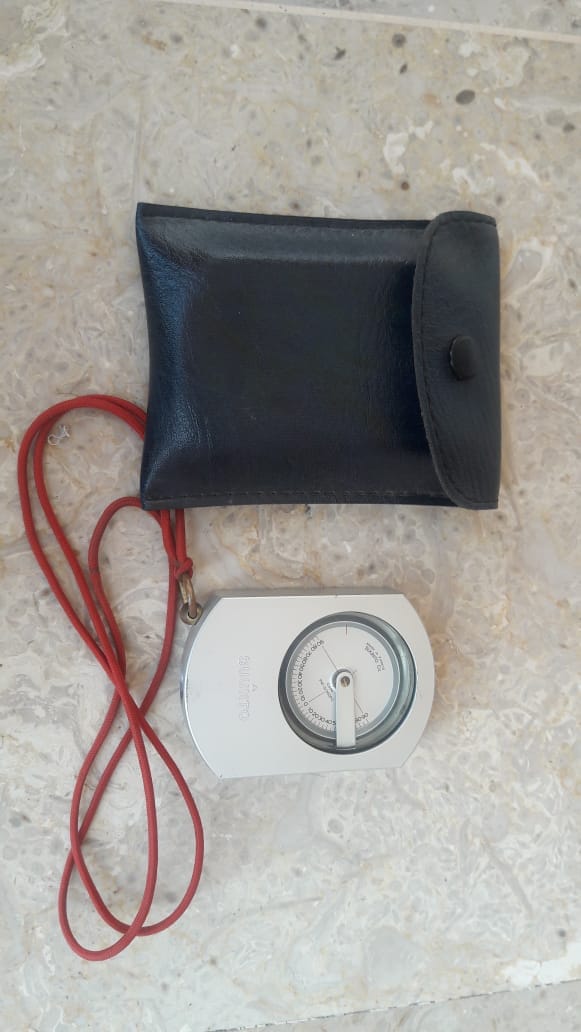

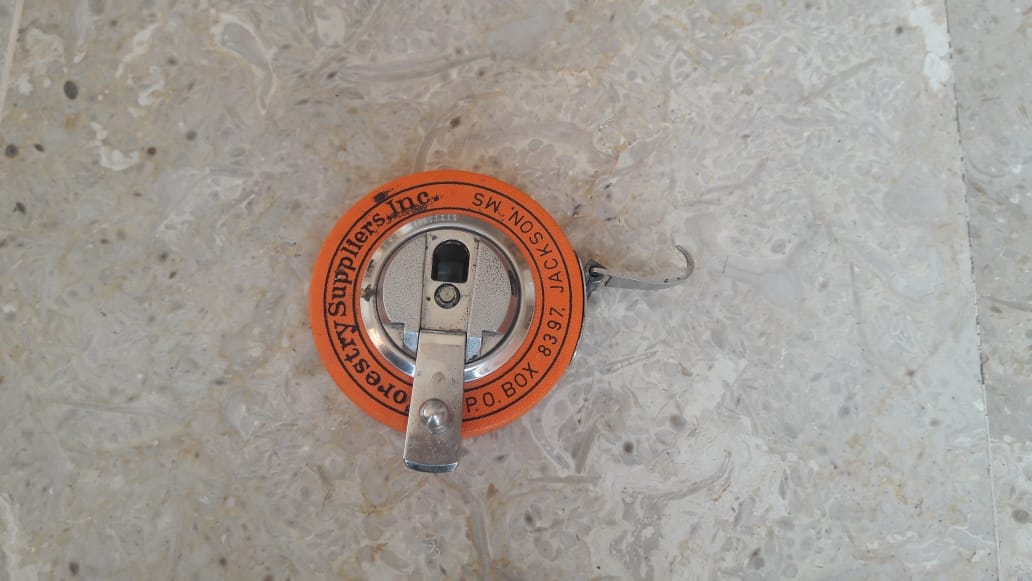


e

c

b

d

c

a


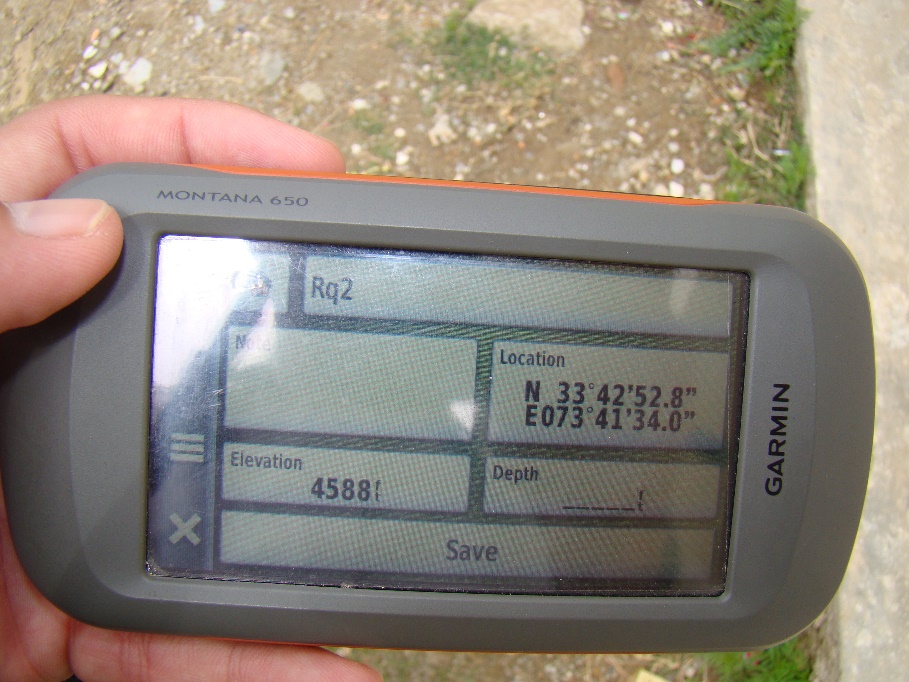

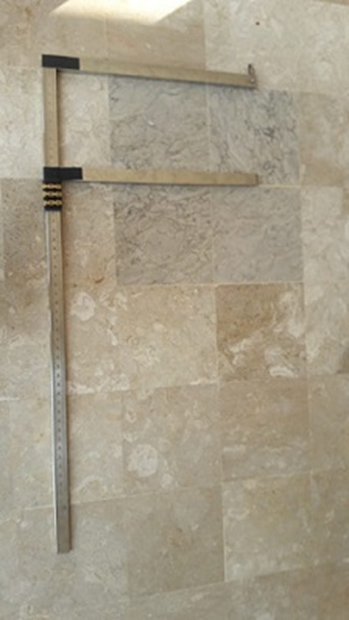


b

d

**Figure S 3: Instruments used in field inventory (a) GPS (b) Abney level (c) Clinometer (d) measuring tap (e) Tree caliper**

e

a

e
