# Supplementary material for: Assessment of forest cover and carbon stock changes in sub-tropical pine forest of Azad Jammu & Kashmir (AJK), Pakistan using multi-temporal Landsat satellite data and field inventory
Source: PLoS One. 2020 Jan 23;15(1):e0226341. doi: 10.1371/journal.pone.0226341 (PMC6977729; doi:10.1371/journal.pone.0226341)
Supplement: S1 File — (PDF) [file pone.0226341.s003.pdf]

Coordinates of field inventory plots and AGB calculation

Plot No.	Latitude (DMS)	Longitude (DMS)	AGB (tons)	Carbon (tons)	AGB (tons/ha)	Carbon (tons/ha)
1	33º 42' 25.9"	73º 40' 25.6"	16	8	160	80
2	33º 42' 23.3"	73º 40' 04.2"	9	4.5	90	45
3	33º 41' 01.5"	73º 39' 36.5"	17	8.5	170	85
4	33º 40' 58.4"	73º 37' 00.1"	24	12	240	120
5	33º 40' 27.7"	73º 36' 58.8"	16	8	160	80
6	33º 39' 10.9"	73º 37' 37.3"	15	7.5	150	75
7	33º 38' 56.6"	73º 37' 20.9"	26	13	260	130
8	33º 38' 09.2"	73º 37' 24.9"	22	11	220	110
9	33º 37' 43.1"	73º 36' 49.2"	8	4	80	40
10	33º 40' 45.4"	73º 37' 55.6"	29	14.5	290	145
11	33º 41' 46.7"	73º 41' 33.3"	12	6	120	60
12	33º 40' 55.5"	73º 42' 19.3"	10	5	100	50
13	33º 40' 22.3"	73º 42' 41.8"	11	5.5	110	55
14	33º 39' 00.7"	73º 43' 28.5"	12	6	120	60
15	33º 38' 43.5"	73º 43' 15.9"	19	9.5	190	95
16	33º 41' 35.9"	73º 42' 39.6"	15	7.5	150	75
17	33º 43' 31.4"	73º 40' 33.8"	14	7	140	70
18	33º 43' 09.0"	73º 39' 28.4"	19	9.5	190	95
19	33º 43' 41.5"	73º 39' 24.5"	26	13	260	130
20	33º 43' 56.2"	73º 39' 013"	27	13.5	270	135
21	33º 44' 23.6"	73º 37' 50.7"	24	12	240	120
22	33º 45' 01.7"	73º 37' 15.7"	28	14	280	140
23	33º 44' 34.3"	73º 36' 27.8"	23	11.5	230	115
24	33º 43' 54.4"	73º 37' 17.3"	14	7	140	70
25	33º 44' 04.1"	73º 40' 08.8"	8	4	80	40
26	33º 44' 39.6"	73º 39' 22.9"	13	6.5	130	65
27	33º 44' 27.8"	73º 39' 34.9"	11	5.5	110	55
28	33º 41' 10.9"	73º 39' 36.0"	5	2.5	50	25
29	33º 41' 17.3"	73º 39' 45.2"	6	3	60	30
30	33º 43' 40.5"	73º 40' 33.3"	8	4	80	40

Plot No.	Latitude (DMS)	Longitude (DMS)	AGB (tons)	Carbon (tons)	AGB (tons/ha)	Carbon (tons/ha)
31	33º 45' 23.6"	73º 40' 53.3"	12	6	120	60
32	33º 46' 08.7"	73º 41' 20.2"	15	7.5	150	75
33	33º 45' 14.7"	73º 41' 41.1"	16	8	160	80
34	33º 45' 14.6"	73º 42' 59.8"	17	8.5	170	85
35	33º 45' 16.0"	73º 41' 00.5"	11	5.5	110	55
36	33º 45' 01.7"	73º 41' 43.9"	16	8	160	80
37	33º 44' 18.8"	73º 41' 03.7"	13	6.5	130	65
38	33º 42' 53.3"	73º 43' 51.6"	9	4.5	90	45
39	33º 43' 59.7"	73º 44' 33.3"	27	13.5	270	135
40	33º 44' 09.6"	73º 44' 47.3"	8	4	80	40
41	33º 42' 59.1"	73º 45' 27.2"	6	3	60	30
42	33º 43' 11.2"	73º 45' 38.1"	12	6	120	60
43	33º 43' 10.8"	73º 45' 56.4"	20	10	200	100
44	33º 43' 28.8"	73º 46' 20.7"	7	3.5	70	35
45	33º 44' 23.3"	73º 47' 11.4"	13	6.5	130	65
46	33º 45' 20.1"	73º 45' 54.0"	12	6	120	60
47	33º 44' 57.6"	73º 47' 10.8"	6	3	60	30
48	33º 45' 41.1"	73º 47' 04.0"	13	6.5	130	65
49	33º 45' 51.2"	73º 46' 32.8"	11	5.5	110	55
50	33º 45' 50.6"	73º 46' 12.8"	8	4	80	40
51	33º 46' 55.8"	73º 43' 27.9"	21	10.5	210	105
52	33º 41' 30.1"	73º 49' 37.7"	9	4.5	90	45
53	33º 41' 28.6"	73º 51' 10.8"	23	11.5	230	115
54	33º 41' 29.1"	73º 52' 01.9"	23	11.5	230	115
55	33º 41' 23.9"	73º 52' 33.0"	30	15	300	150
56	33º 41' 24.8"	73º 48' 52.9"	7	3.5	70	35
57	33º 39' 31.2"	73º 48' 21.0"	11	5.5	110	55
58	33º 37' 30.9"	73º 49' 31.5"	3	1.5	30	15
59	33º 41' 03.2"	73º 48' 43.0"	17	8.5	170	85
60	33º 41' 41.3"	73º 50' 12.0"	28	14	280	140
61	33º 43' 03.5"	73º 50' 16.5"	5	2.5	50	25
62	33º 43' 47.7"	73º 49' 27.2"	5	2.5	50	25

Plot No.	Latitude (DMS)	Longitude (DMS)	AGB (tons)	Carbon (tons)	AGB (tons/ha)	Carbon (tons/ha)
63	33º 45' 26.9"	73º 47' 43.8"	18	9	180	90
64	33º 46' 22.6"	73º 47' 45.1"	15	7.5	150	75
65	33º 46' 22.6"	73º 48' 34.6"	13	6.5	130	65
66	33º 47' 09.6"	73º 46' 50.6"	14	7	140	70
67	33º 46' 05.1"	73º 47' 06.5"	16	8	160	80
68	33º 45' 44.8"	73º 46' 54.8"	10	5	100	50
69	33º 46' 42.4"	73º 45' 33.2"	18	9	180	90
70	33º 46' 58.3"	73º 44' 31.4"	5	2.5	50	25
71	33º 46' 50.6"	73º 42' 42.4"	13	6.5	130	65
72	33º 44' 50.9"	73º 37' 02.4"	23	11.5	230	115
73	33º 46' 59.2"	73º 37' 46.1"	5	2.5	50	25
74	33º 47' 39.5"	73º 37' 55.5"	15	7.5	150	75
75	33º 48' 42.7"	73º 37' 25.7"	12	6	120	60
76	33º 49' 27.4"	73º 37' 46.3"	19	9.5	190	95
77	33º 49' 55.7"	73º 38' 24.4"	17	8.5	170	85
78	33º 49' 41.7"	73º 38' 13.9"	15	7.5	150	75
79	33º 49' 31.5"	73º 37' 31.2"	13	6.5	130	65
80	33º 44' 25.0"	73º 38' 31.4"	27	13.5	270	135
81	33º 41' 32.8"	73º 38' 107"	22	11	220	110
82	33º 42' 55.0"	73º 42 ' 667"	6	3	60	30
83	33º 46' 77.9"	73º 42' 825"	14	7	140	70
84	33º 45' 37.9"	73º 42' 41.5"	12	6	120	60
85	33º 41' 79.8"	73º 41' 30.4"	6	3	60	30
86	33º 43' 44.6"	73º 41' 14.8"	13	6.5	130	65
87	33º 38' 49.6"	73º 43' 42.5"	23	11.5	230	115
88	33º 38' 29.8"	73º 44' 43.1"	7	3.5	70	35
89	33º 38' 26.1"	73º 45' 12.6"	13	6.5	130	65
90	33º 39' 45.3"	73º 44' 49.0"	10	5	100	50
91	33º 38' 46.6"	73º 47' 05.3"	20	10	200	100
92	33º 39' 20.9"	73º 46' 25.9"	22	11	220	110
93	33º 40' 15.2"	73º 45' 23.6"	13	6.5	130	65
94	33º 40' 10.9"	73º 44' 51.0"	8	4	80	40

Plot No.	Latitude (DMS)	Longitude (DMS)	AGB (tons)	Carbon (tons)	AGB (tons/ha)	Carbon (tons/ha)
95	33º 38' 42.5"	73º 44' 59.1"	9	4.5	90	45
96	33º 38' 40.6"	73º 44' 21.2"	15	7.5	150	75
97	33º 39' 50.9"	73º 39' 56.1"	9	4.5	90	45
98	33º 39' 14.8"	73º 39' 50.4"	12	6	120	60
99	33º 39' 45.3"	73º 40' 0.5"	7	3.5	70	35
100	33º 39' 58.7"	73º 40' 04.4"	5	2.5	50	25
101	33º 42' 09.0"	73º 38' 32.2"	11	5.5	110	55
102	33º 42' 10.3"	73º 39' 59.9"	9	4.5	90	45
103	33º 43' 16.8"	73º 37' 22.8"	14	7	140	70
104	33º 43' 52.5"	73º 37' 50.5"	26	13	260	130
105	33º 44' 39.5"	73º 37' 06.6"	25	12.5	250	125
106	33º 44' 47.6"	73º 37' 31.2"	25	12.5	250	125
107	33º 44' 47.8"	73º 37' 58.2"	9	4.5	90	45
108	33º 43' 36.2"	73º 41' 09.9"	15	7.5	150	75
