# Supplementary material for: Assessment of forest cover and carbon stock changes in sub-tropical pine forest of Azad Jammu & Kashmir (AJK), Pakistan using multi-temporal Landsat satellite data and field inventory
Source: PLoS One. 2020 Jan 23;15(1):e0226341. doi: 10.1371/journal.pone.0226341 (PMC6977729; doi:10.1371/journal.pone.0226341)
Supplement: S1 Table — (DOCX) [file pone.0226341.s004.docx]

S1 Table: Error matrix for Landsat OLI 2018 image classification

| **Classified image** | **Reference Data** | | | |
| --- | --- | --- | --- | --- |
|  |  | Forest | Non Forest | **Row Total** |
|  | Forest | 77 | 3 | 80 |
|  | Non Forest | 2 | 58 | 60 |
|  | **Column Total** | 79 | 61 | 140 |

**Overall Accuracy= 135/ 140= 96%; Kappa coefficient: 0.92**

**Producer’s Accuracy** **User’s Accuracy**

Forest = 77/79 =97% Forest = 77/80= 96%

Non Forest = 58/61= 95% Non Forest = 58/60= 97%
